# Comprehensive Evaluation of Anti-PD-1, Anti-PD-L1, Anti-CTLA-4 and Their Combined Immunotherapy in Clinical Trials: A Systematic Review and Meta-analysis

**DOI:** 10.3389/fphar.2022.883655

**Published:** 2022-05-25

**Authors:** Ze Xiang, Jiayuan Li, Zhengyu Zhang, Chao Cen, Wei Chen, Bin Jiang, Yiling Meng, Ying Wang, Björn Berglund, Guanghua Zhai, Jian Wu

**Affiliations:** ^1^ Zhejiang University School of Medicine, Hangzhou, China; ^2^ Center for Global Health, Department of Epidemiology, School of Public Health, Nanjing Medical University, Nanjing, China; ^3^ Department of Hepatobiliary and Pancreatic Surgery, The First Affiliated Hospital, Zhejiang University School of Medicine, Hangzhou, China; ^4^ Key Laboratory of Cancer Prevention and Therapy Combining Traditional Chinese and Western Medicine of Zhejiang Province, Tongde Hospital of Zhejiang Province, Cancer Institute of Integrated Traditional Chinese and Western Medicine, Zhejiang Academy of Traditional Chinese Medicine, Hangzhou, China; ^5^ Department of Laboratory Medicine, The Central Blood Station of Yancheng City, Yancheng, China; ^6^ Department of Laboratory Medicine, Suzhou Vocational Health College, Suzhou, China; ^7^ Department of Clinical Laboratory, Suzhou Municipal Hospital, Gusu School, The Affiliated Suzhou Hospital of Nanjing Medical University, Nanjing Medical University, Suzhou, China; ^8^ Department of Biomedical and Clinical Sciences, Linköping University, Linköping, Sweden

**Keywords:** immune checkpoint inhibitor, cancer immunotherapy, programmed death-1 (PD-1), programmed death-ligand-1 (PD-L1), cytotoxic T lymphocyte antigen-4 (CTLA-4)

## Abstract

Immunotherapy with immune checkpoint inhibitor (ICI) drugs is gradually becoming a hot topic in cancer treatment. To comprehensively evaluate the safety and efficacy of ICI drugs, we employed the Bayesian model and conducted a network meta-analysis in terms of progression-free survival (PFS), overall survival (OS) and severe adverse events (AEs). Our study found that treatment with ipilimumab was significantly worse than standard therapies in terms of PFS, whereas treatment with cemiplimab significantly improved PFS. The results also indicated that cemiplimab was the best choice for PFS. Treatment with nivolumab, pembrolizumab and nivolumab plus ipilimumab significantly improved OS compared to standard therapies. In terms of OS, cemiplimab was found to be the best choice, whereas avelumab was the worst. In terms of severe AEs, atezolizumab, avelumab, durvalumab, nivolumab, and pembrolizumab all significantly reduced the risk of grade 3 or higher AEs compared to standard therapy. The least likely to be associated with severe AEs were as follows: cemiplimab, avelumab, nivolumab, atezolizumab, and camrelizumab, with nivolumab plus ipilimumab to be the worst. Therefore, different ICI drug therapies may pose different risks in terms of PFS, OS and severe AEs. Our study may provide new insights and strategies for the clinical practice of ICI drugs.

## 1 Introduction

Immunotherapy has become one of the most important breakthroughs in the treatment of cancer in recent years, and its development has promoted changes in many cancer treatment methods. As a series of co-inhibitory and co-stimulatory receptors and ligands, immune checkpoint inhibitors (ICI) drugs can block negative regulatory factors expressed by immune or tumor cells to enhance their immune function against cancer cells, mainly programmed death-1 (PD-1), programmed death-ligand-1 (PD-L1) and cytotoxic T lymphocyte antigen-4 (CTLA-4) (Rosenberg et al., 2004). In 2011, the CTLA-4 inhibitor ipilimumab was approved by the US Food and Drug Administration for the treatment of advanced melanoma (Hodi et al., 2010). Subsequently, several ICI drugs were also approved for the treatment of cancer (Topalian et al., 2012; Gong et al., 2018). Since then, interest for immunotherapy with ICI drugs has been increasing. Many studies focused on the prognosis and treatment for different cancers (Wu et al., 2015).

Chemotherapy is the first-line treatment for advanced cancer, and patients undergoing chemotherapy often experience severe adverse events (AEs). Although ICI drugs have achieved good anticancer effects in the treatment of many solid tumors, they may still cause severe treatment-related or drug-related AEs. Progression-free survival (PFS) and overall survival (OS) are usually efficacy end-points. In terms of PFS and OS, the therapeutic effects of ICI drugs remain unclear compared with standard therapies. Due to the limitations of randomized clinical trials, the overall safety evaluation of different ICI drugs for cancer treatment is not comprehensive, especially in terms of PFS and OS.

We conducted a systematic review and network meta-analysis of the therapeutic effects of ICI drugs targeting PD-1, PD-L1, and CTLA-4, focusing on PFS, OS and treatment-related severe AEs in patients receiving ICI drug monotherapy, combination therapies and standard therapies (chemotherapy, targeted therapies and their combination therapies included). This study comprehensively evaluated the safety and efficacy of different ICI drugs and their combination therapies, aiming to provide better guidance for the clinical application of various ICI drugs.

## 2 Methods

### 2.1 Search Methods and Study Selection

We searched PubMed, Embase, and Cochrane Library for English-language studies between January 2000 and September 2021, using keywords such as ipilimumab, tremelimumab, pembrolizumab, nivolumab, cemiplimab, camrelizumab, toripalimab, tislelizumab, spartalizumab, atezolizumab, avelumab, durvalumab, PD-1, PD-L1, and CTLA-4. The search strategy was described in Supplementary Table S1. The study search, selection and data extraction were independently conducted by two reviewers (ZX and ZZ), and discrepancies were evaluated by an independent reviewer (JL). The three authors (ZX, JL and ZZ) reviewed and discussed the full text of studies that may be eligible, and differences of opinions were resolved by consensus.

Only high-quality head-to-head phase 2 and 3 randomized controlled trials (RCTs) comparing two or more treatments including ICI drug monotherapy, ICI drug combination therapies and standard therapies were included. Some RCTs only presented interim results, as insufficient information may affect the final analysis, we selected the most recent results as much as possible. Data provided include at least one of the following: hazard ratios (HRs) of PFS, OS and treatment-related severe AEs. We excluded reviews, conference abstracts and posters. The study was performed based on the Preferred Reporting Items for Systematic Reviews and Meta-analyses (PRISMA) guideline (Hutton et al., 2015; Wang et al., 2021). This study was approved by International prospective register of systematic reviews (PROSPERO) (registered ID: CRD42021278158).

### 2.2 Data Extraction

The authors (ZX and ZZ) independently extracted data according to the PRISMA guidelines. The first author, year of publication, national clinical trial identification number, trial name, phase, number of patients, type of cancer, drug used, follow-up time, number of severe AEs, HRs, and confidence interval (CI) of PFS and OS were summarized in standardized Tables 1–3.

**TABLE 1:** List of the studies involving PFS in this meta-analysis.

First author	Year	NCT	Trial name	Total number	Phase	Canner type	Treatment 1	Patient number	Treatment 2	Patient number	Follow-up time	PFS HR	PFS CI lower limit	PFS CI upper limit
Fehrenbacher L et al. (Fehrenbacher et al., 2018)	2018	NCT02008227	OAK	1225	3	Non small cell lung cancer	Atezolizumab	613	Docetaxel	612	21	0.96	0.85	1.08
McDermott DF et al. (McDermott et al., 2018)	2018	NCT01984242	IMmotion150	204	2	Renal cell carcinoma	Atezolizumab	103	Sunitinib	101	20.7	1.19	0.82	1.71
Powles T et al. (Powles et al., 2018)	2018	NCT02302807	IMvigor211	234	3	Urothelial carcinoma	Atezolizumab	116	Chemotherapy	118	17.3	1.01	0.75	1.34
Eng C et al. (Eng et al., 2019)	2019	NCT02788279	IMblaze370	180	3	Colorectal cancer	Atezolizumab	90	Regorafenib	90	7.3	1.39	1.00	1.94
Pujol JL et al. (Pujol et al., 2019)	2019	NCT03059667	IFCT-1603	73	2	Small Cell Lung Cancer	Atezolizumab	49	Chemotherapy	24	13.7	2.26	1.3	3.93
Herbst RS et al. (Herbst et al., 2020)	2020	NCT02409342	IMpower110	554	3	Non small cell lung cancer	Atezolizumab	277	Chemotherapy	277	13.4	0.77	0.63	0.94
Bang YJ et al. (Bang et al., 2018)	2018	NCT02625623	JAVELIN Gastric 300	371	3	Gastric/gastrooesophageal junction cancer	Avelumab	185	Chemotherapy	186	10.6	1.73	1.4	2.2
Barlesi F et al. (Barlesi et al., 2018)	2018	NCT02395172	JAVELIN Lung 200	529	3	Non small cell lung cancer	Avelumab	264	Docetaxel	265	T1:18.9	1.01	0.80	1.28
											T2:17.8			
Pujade-Lauraine E et al. (Pujade-Lauraine et al., 2021)	2021	NCT02580058	JAVELIN Ovarian 200	378	3	Ovarian cancer	Avelumab	188	Pegylated liposomal doxorubicin	190	T1:18.2	1.68	1.32	2.60
											T2:17.4			
Huang J et al. (Huang et al., 2020)	2020	NCT03099382	ESCORT	448	3	Squamous cell carcinoma	Camrelizumab	228	Chemotherapy	220	8.3	0.69	0.56	0.86
Sezer A et al. (Sezer et al., 2021)	2021	NCT03088540	EMPOWER-Lung 1	563	3	Non small cell lung cancer	Cemiplimab	283	Chemotherapy	280	T1:10.8	0.54	0.43	0.68
											T2:10.9			
Siu LL et al. (Siu et al., 2019)	2019	NCT02319044	CONDOR	267	2	Squamous cell carcinoma	Durvalumab+	133	Durvalumab	67	T1:6.5	1.13	0.82	1.56
							Tremelimumab				T2:6.0			
					2	Squamous cell carcinoma	Durvalumab+	133	Tremelimumab	67	T1:6.5	0.73	0.53	1.01
							Tremelimumab				T2:5.2			
Ferris RL et al. (Ferris et al., 2020)	2020	NCT02369874	EAGLE	736	3	Squamous cell carcinoma	Durvalumab	240	Standard of care	249	T1:7.6	1.02	0.84	1.25
											T2:7.8			
					3	Squamous cell carcinoma	Durvalumab+	247	Standard of care	249	T1:6.3	1.09	0.90	1.33
							Tremelimumab				T2:7.8			
Planchard D et al. (Planchard et al., 2020)	2020	NCT02352948	ARCTIC	595	3	Non small cell lung cancer	Durvalumab	62	Standard of care	64	9.1	0.71	0.49	1.04
					3	Non small cell lung cancer	Durvalumab+	174	Standard of care	118	9.1	0.77	0.59	1.01
							Tremelimumab							
					3	Non small cell lung cancer	Durvalumab	117	Standard of care	118	9.1	0.87	0.65	1.16
					3	Non small cell lung cancer	Tremelimumab	60	Standard of care	118	9.1	1.25	0.88	1.77
Rizvi NA et al. (Rizvi et al., 2020)	2020	NCT02453282	MYSTIC	488	3	Non small cell lung cancer	Durvalumab	163	Chemotherapy	162	10.6	0.87	0.59	1.29
					3	Non small cell lung cancer	Durvalumab+	163	Chemotherapy	162	10.6	1.05	0.72	1.53
							Tremelimumab							
Bachelot T et al. (Bachelot et al., 2021)	2021	NCT02299999	SAFIR02-BREAST IMMUNO	199	2	Breast cancer	Durvalumab	68	Chemotherapy	131	19.7	1.40	1.00	1.96
Bang YJ et al. (Bang et al., 2017)	2017	NCT01585987	NA	108	2	Gastric/gastrooesophageal junction cancer	Ipilimumab	57	Best supportive care	51	24	1.44	1.09	1.91
Borghaei H et al. (Borghaei et al., 2015)	2015	NCT01673867	CheckMate 057	582	3	Non small cell lung cancer	Nivolumab	292	Docetaxel	290	13.2	0.92	0.77	1.11
Brahmer J et al. (Brahmer et al., 2015)	2015	NCT01642004	CheckMate 017	272	3	Non small cell lung cancer	Nivolumab	135	Docetaxel	137	11	0.62	0.47	0.81
Motzer RJ et al. (Motzer et al., 2015)	2015	NCT01668784	CheckMate 025	821	3	Renal cell carcinoma	Nivolumab	410	Everolimus	411	14	0.88	0.75	1.03
Ferris RL et al. (Ferris et al., 2016)	2016	NCT02105636	CheckMate 141	361	3	Squamous cell carcinoma	Nivolumab	240	Standard therapy	121	5.1	0.89	0.70	1.13
Hodi FS et al. (Hodi et al., 2016)	2016	NCT01927419	CheckMate 069	142	2	Melanoma	Nivolumab+	95	Ipilimumab	47	24.5	0·36	0.22	0.56
							Ipilimumab							
Carbone DP et al. (Carbone et al., 2017)	2017	NCT02041533	CheckMate 026	541	3	Non small cell lung cancer	Nivolumab	271	Chemotherapy	270	13.5	1.19	0.97	1.46
Hodi FS et al. (Hodi et al., 2018)	2018	NCT01844505	CheckMate 067	945	3	Melanoma	Nivolumab+	314	Ipilimumab	315	T1：46.9	0.42	0.35	0.51
							Ipilimumab	T2:18.6						
					3	Melanoma	Nivolumab	316	Ipilimumab	315	T1:18.6	0.53	0.44	0.64
											T2:36			
Larkin J et al. (Larkin et al., 2018)	2018	NCT01721746	CheckMate 037	405	3	Melanoma	Nivolumab	272	Chemotherapy	133	24	1.00	0.78	1.44
Hellmann MD et al. (Hellmann et al., 2019)	2019	NCT02477826	CheckMate 227	299	3	Non small cell lung cancer	Nivolumab+	139	Chemotherapy	160	11.2	0.58	0.41	0.81
							Ipilimumab							
Kato K et al. (Kato et al., 2019)	2019	NCT02569242	ATTRACTION-3	419	3	Squamous cell carcinoma	Nivolumab	210	Chemotherapy	209	17.6	1.08	0.87	1.34
Wu YL et al. (Wu et al., 2019)	2019	NCT02613507	CheckMate 078	504	3	Non small cell lung cancer	Nivolumab	338	Docetaxel	166	8.8	0.77	0.62	0.95
Motzer RJ et al. (Motzer et al., 2020)	2020	NCT02231749	CheckMate 214	1096	3	Renal cell carcinoma	Nivolumab+	550	Sunitinib	546	42	0.88	0.75	1.04
							Ipilimumab							
Reardon DA et al. (Reardon et al., 2020)	2020	NCT02017717	CheckMate 143	369	3	Glioblastoma	Nivolumab	184	Bevacizumab	185	9.5	1.97	1.57	2.48
Robert C et al. (Robert et al., 2020)	2020	NCT01721772	CheckMate 066	418	3	Melanoma	Nivolumab	210	Dacarbazine	208	60	0.40	0.33	0.54
Zamarin D et al. (Zamarin et al., 2020)	2020	NCT02498600	NRG GY003	100	2	Ovarian Cancer	Nivolumab+	51	Nivolumab	49	NA	0.53	0.34	0.82
							Ipilimumab							
Baas P et al. (Baas et al., 2021)	2021	NCT02899299	CheckMate 743	605	3	Malignant pleural mesothelioma	Nivolumab+	303	Chemotherapy	302	29.7	1.00	0.82	1.21
							Ipilimumab							
Spigel DR et al. (Spigel et al., 2021)	2021	NCT02481830	CheckMate 331	569	3	Small cell lung cancer	Nivolumab	284	Chemotherapy	285	15.8	1.41	1.18	1.69
Tannir NM et al. (Tannir et al., 2021)	2021	NA	CheckMate 214	139	3	Renal cell carcinoma	Nivolumab+	74	Sunitinib	65	42	0.54	0.33	0.86
							Ipilimumab							
Herbst RS et al. (Herbst et al., 2016)	2016	NCT01905657	KEYNOTE-010	687	2/3	Non small cell lung cancer	Pembrolizumab	344	Docetaxel	343	13.1	0.88	0.74	1.05
Hamid O et al. (Hamid et al., 2017)	2017	NCT01704287	KEYNOTE-002	359	2	Melanoma	Pembrolizumab	180	Chemotherapy	179	28	0.58	0.46	0.73
Shitara K et al. (Shitara et al., 2018)	2018	NCT02370498	KEYNOTE-061	395	3	Gastric/gastrooesophageal junction cancer	Pembrolizumab	196	Paclitaxel	199	8.5	1.27	1.03	1.57
Cohen EEW et al. (Cohen et al., 2019)	2019	NCT02252042	KEYNOTE-040	495	3	Ssquamous cell carcinoma	Pembrolizumab	247	Standard of care	248	T1:7.5	0.96	0.79	1.16
											T2:7.1			
Fradet Y et al. (Fradet et al., 2019)	2019	NCT02256436	KEYNOTE-045	542	3	Urothelial cancer	Pembrolizumab	270	Chemotherapy	272	27.7	0.96	0.79	1.16
Mok TSK et al. (Mok et al., 2019)	2019	NCT02220894	KEYNOTE-042	1274	3	Non small cell lung cancer	Pembrolizumab	637	Chemotherapy	637	12.8	1.07	0.94	1.21
Reck M et al. (Reck et al., 2019)	2019	NCT02142738	KEYNOTE-024	305	3	Non small cell lung cancer	Pembrolizumab	154	Chemotherapy	151	11.2	0.50	0.37	0.68
Robert C et al. (Robert et al., 2019)	2019	NCT01866319	KEYNOTE-006	834	3	Melanoma	Pembrolizumab	556	Ipilimumab	278	57.7	0.57	0.48	0.67
André T et al. (André et al., 2020)	2020	NCT02563002	KEYNOTE-177	307	3	Colorectal cancer	Pembrolizumab	153	Chemotherapy	154	32.4	0.60	0.45	0.80
Kojima T et al. (Kojima et al., 2020)	2020	NCT02564263	KEYNOTE-181	628	3	Esophageal Cancer	Pembrolizumab	314	Chemotherapy	314	T1:7.1	1.11	0.94	1.31
											T2:6.9			
Popat S et al. (Popat et al., 2020)	2020	NCT02991482	ETOP 9-15	144	3	Malignant pleural mesothelioma	Pembrolizumab	73	Chemotherapy	71	17.5	1.06	0.73	1.53
Shitara K et al. (Shitara et al., 2020)	2020	NCT02494583	KEYNOTE-062	506	3	Gastric/gastrooesophageal junction cancer	Pembrolizumab	256	Chemotherapy	250	29.4	1.66	1.37	2.01
Kuruvilla J et al. (Kuruvilla et al., 2021)	2021	NCT02684292	KEYNOTE-204	304	3	Hodgkin lymphoma	Pembrolizumab	151	Brentuximab vedotin	153	24	0.65	0.48	0.88

PFS = Progression-free survival. HR = Hazard ratio. CI = Confidence interval.

**TABLE 2:** List of the studies involving OS in this meta-analysis.

First author	Year	NCT	Trial name	Total number	Phase	Canner type	Treatment 1	Patient number	Treatment 2	Patient number	Follow-up time	OS HR	OS CI lower limit	OS CI upper limit
Fehrenbacher L et al. (Fehrenbacher et al., 2016)	2016	NCT01903993	POPLAR	287	2	Non small cell lung cancer	Atezolizumab	144	Docetaxel	143	13	0.73	0.53	0.99
Fehrenbacher L et al. (Fehrenbacher et al., 2018)	2018	NCT02008227	OAK	1225	3	Non small cell lung cancer	Atezolizumab	613	Docetaxel	612	26	0.80	0.70	0.92
Powles T et al. (Powles et al., 2018)	2017	NCT02302807	IMvigor211	234	3	Urothelial carcinoma	Atezolizumab	116	Chemotherapy	118	17.3	0.87	0.63	1.21
Eng C et al. (Eng et al., 2019)	2019	NCT02788279	IMblaze370	180	3	Colorectal cancer	Atezolizumab	90	Regorafenib	90	7.3	1.19	0.83	1.71
Pujol JL et al. (Pujol et al., 2019)	2018	NCT03059667	IFCT-1603	73	2	Small cell lung cancer	Atezolizumab	49	Chemotherapy	24	13.7	0.84	0.45	1.58
Herbst RS et al. (Herbst et al., 2020)	2020	NCT02409342	IMpower110	554	3	Non small cell lung cancer	Atezolizumab	277	Chemotherapy	277	13.4	0.83	0.65	1.07
Bang YJ er al (Bang et al., 2018)	2018	NCT02625623	JAVELIN Gastric 300	371	3	Gastric/gastrooesophageal junction cancer	Avelumab	185	Chemotherapy	186	10.6	1.1	0.9	1.4
Park K et al. (Park et al., 2021)	2021	NCT02395172	JAVELIN Lung 200	529	3	Non small cell lung cancer	Avelumab	264	Docetaxel	265	24	0.87	0.71	1.05
Pujade-Lauraine E et al. (Pujade-Lauraine et al., 2021)	2021	NCT02580058	JAVELIN Ovarian 200	378	3	Ovarian cancer	Avelumab	188	Pegylated liposomal doxorubicin	190	T1:18.2	1.14	0.95	1.58
											T2:17.4			
Huang J et al. (Huang et al., 2020)	2020	NCT03099382	ESCORT	448	3	Squamous cell carcinoma	Camrelizumab	228	Chemotherapy	220	8.3	0.71	0.57	0.87
Sezer A et al. (Sezer et al., 2021)	2021	NCT03088540	EMPOWER-Lung 1	563	3	Non small cell lung cancer	Cemiplimab	283	Chemotherapy	280	T1:10.8	0.57	0.42	0.77
											T2:10.9			
Siu LL et al. (Siu et al., 2019)	2019	NCT02319044	CONDOR	267	2	Squamous cell carcinoma	Durvalumab+	133	Durvalumab	67	T1:6.5	0.99	0.69	1.43
							Tremelimumab				T2:6.0			
					2	Squamous cell carcinoma	Durvalumab+	133	Tremelimumab	67	T1:6.5	0.72	0.51	1.03
							Tremelimumab				T2:5.2			
Ferris RL et al. (Ferris et al., 2020)	2020	NCT02369874	EAGLE	736	3	Squamous cell carcinoma	Durvalumab	240	Standard of care	249	T1:7.6 T2:7.8	0.88	0.72	1.08
					3	Squamous cell carcinoma	Durvalumab+	247	Standard of care	249	T1:6.3	1.04	0.85	1.26
							Tremelimumab				T2:7.8			
Planchard D et al. (Planchard et al., 2020)	2020	NCT02352948	ARCTIC	595	3	Non small cell lung cancer	Durvalumab	62	Standard of care	64	9.1	0.63	0.42	0.93
					3	Non small cell lung cancer	Durvalumab+ Tremelimumab	174	Standard of care	118	9.1	0.80	0.61	1.05
					3	Non small cell lung cancer	Durvalumab	117	Standard of care	118	9.1	0.80	0.59	1.08
					3	Non small cell lung cancer	Tremelimumab	60	Standard of care	118	9.1	1.02	0.71	1.46
Powles T et al. (Powles et al., 2020)	2020	NCT02516241	DANUBE	1032	3	Urothelial carcinoma	Durvalumab	346	Chemotherapy	344	41.2	0.99	0.83	1.17
					3	Urothelial carcinoma	Durvalumab+	342	Chemotherapy	344	41.2	0.85	0.72	1.02
							Tremelimumab							
Rizvi NA et al. (Rizvi et al., 2020)	2020	NCT02453282	MYSTIC	488	3	Non small cell lung cancer	Durvalumab	163	Chemotherapy	162	30.2	0.76	0.56	1.02
					3	Non small cell lung cancer	Durvalumab+	163	Chemotherapy	162	30.2	0.85	0.61	1.17
							Tremelimumab							
Bachelot T et al. (Bachelot et al., 2021)	2021	NCT02299999	SAFIR02-BREAST IMMUNO	199	2	Breast cancer	Durvalumab	68	Chemotherapy	131	19.7	0.84	0.54	1.29
Hodi FS et al. (Hodi et al., 2010)	2010	NCT00094653	MDX010-20	273	3	Melanoma	Ipilimumab	137	Gp100	136	T1:27.8 T2:17.2	0.66	0.51	0.87
Tarhini AA et al. (Tarhini et al., 2020)	2020	NA	E1609	1159	3	Melanoma	Ipilimumab	523	Interferon Alfa-2b	636	57.4	0.78	0.61	0.99
Borghaei H et al. (Borghaei et al., 2015)	2015	NCT01673867	CheckMate 057	582	3	Non small cell lung cancer	Nivolumab	292	Docetaxel	290	13.2	0.73	0.59	0.89
Brahmer J et al. (Brahmer et al., 2015)	2015	NCT01642004	CheckMate 017	272	3	Non small cell lung cancer	Nivolumab	135	Docetaxel	137	11	0.59	0.44	0.79
Motzer RJ et al. (Motzer et al., 2015)	2015	NCT01668784	CheckMate 025	821	3	Renal cell carcinoma	Nivolumab	410	Everolimus	411	14	0.73	0.57	0.93
Ferris RL et al. (Ferris et al., 2016)	2016	NCT02105636	CheckMate 141	361	3	Squamous cell carcinoma	Nivolumab	240	Standard therapy	121	5.1	0.70	0.51	0.96
Hodi FS et al. (Hodi et al., 2016)	2016	NCT01927419	CheckMate 069	142	2	Melanoma	Nivolumab+	95	Ipilimumab	47	24.5	0.74	0.43	1.26
							Ipilimumab							
Carbone DP et al. (Carbone et al., 2017)	2017	NCT02041533	CheckMate 026	541	3	Non small cell lung cancer	Nivolumab	271	Chemotherapy	270	13.5	1.08	0.87	1.34
Hodi FS et al. (Hodi et al., 2018)	2018	NCT01844505	CheckMate 067	945	3	Melanoma	Nivolumab+ Ipilimumab	314	Ipilimumab	315	T1:46.9 T2:18.6	0.54	0.44	0.67
					3	Melanoma	Nivolumab	316	Ipilimumab	315	T1: 36 T2:18.6	0.65	0.53	0.79
Larkin J et al. (Larkin et al., 2018)	2018	NCT01721746	CheckMate 037	405	3	Melanoma	Nivolumab	272	Chemotherapy	133	24	0.95	0.73	1.24
Hellmann MD et al. (Hellmann et al., 2019)	2019	NCT02477826	CheckMate 227	1166	3	Non small cell lung cancer	Nivolumab+ Ipilimumab	583	Chemotherapy	583	29.3	0.73	0.64	0.84
Kato K et al. (Kato et al., 2019)	2019	NCT02569242	ATTRACTION-3	419	3	Squamous cell carcinoma	Nivolumab	210	Chemotherapy	209	17.6	0.77	0.62	0.96
Wu YL et al. (Wu et al., 2019)	2019	NCT02613507	CheckMate 078	504	3	Non small cell lung cancer	Nivolumab	338	Docetaxel	166	8.8	0.68	0.52	0.90
Motzer RJ et al. (Motzer et al., 2020)	2020	NCT02231749	CheckMate 214	1096	3	Renal cell carcinoma	Nivolumab+	550	Sunitinib	546	42	0.72	0.61	0.86
							Ipilimumab							
Reardon DA et al. (Reardon et al., 2020)	2020	NCT02017717	CheckMate 143	369	3	Glioblastoma	Nivolumab	184	Bevacizumab	185	9.5	1.04	0.83	1.3
Robert C et al. (Robert et al., 2020)	2020	NCT01721772	CheckMate 066	418	3	Melanoma	Nivolumab	210	Dacarbazine	208	60	0.50	0.40	0.63
Zamarin D et al. (Zamarin et al., 2020)	2020	NCT02498600	NRG GY003	100	2	Ovarian cancer	Nivolumab+	51	Nivolumab	49	NA	0.79	0.44	1.42
							Ipilimumab							
Baas P et al. (Baas et al., 2021)	2021	NCT02899299	CheckMate 743	605	3	Malignant pleural mesothelioma	Nivolumab+	303	Chemotherapy	302	29.7	0.74	0.60	0.91
							Ipilimumab							
Spigel DR et al. (Spigel et al., 2021)	2021	NCT02481830	CheckMate 331	569	3	Small cell lung cancer	Nivolumab	284	Chemotherapy	285	15.8	0.86	0.72	1.04
Tannir NM et al. (Tannir et al., 2021)	2021	NA	CheckMate 214	139	3	Renal cell carcinoma	Nivolumab+	74	Sunitinib	65	42	0.45	0.30	0.70
							Ipilimumab							
Ribas A et al. (Ribas et al., 2013)	2013	NCT00257205	NA	655	3	Melanoma	Tremelimumab	328	Chemotherapy	327	NA	0.88	NA	NA
Herbst RS et al. (Herbst et al., 2016)	2016	NCT01905657	KEYNOTE-010	687	2/3	Non small cell lung cancer	Pembrolizumab	344	Docetaxel	343	13.1	0.71	0.58	0.88
Hamid O et al. (Hamid et al., 2017)	2017	NCT01704287	KEYNOTE-002	359	2	Melanoma	Pembrolizumab	180	Chemotherapy	179	28	0.86	0.67	1.10
Shitara K et al. (Shitara et al., 2018)	2018	NCT02370498	KEYNOTE-061	395	3	Gastric/gastrooesophageal junction cancer	Pembrolizumab	196	Paclitaxel	199	8.5	0.82	0.66	1.03
Cohen EEW et al. (Cohen et al., 2019)	2019	NCT02252042	KEYNOTE-040	495	3	Squamous cell carcinoma	Pembrolizumab	247	Standard of care	248	7.5	0.80	0.65	0.98
Fradet Y et al. (Fradet et al., 2019)	2019	NCT02256436	KEYNOTE-045	542	3	Urothelial cancer	Pembrolizumab	270	Chemotherapy	272	27.7	0.70	0.57	0.85
Mok TSK et al. (Mok et al., 2019)	2019	NCT02220894	KEYNOTE-042	1274	3	Non small cell lung cancer	Pembrolizumab	637	Chemotherapy	637	12.8	0.81	0.71	0.93
Reck M et al. (Reck et al., 2019)	2019	NCT02142738	KEYNOTE-024	305	3	Non small cell lung cancer	Pembrolizumab	154	Chemotherapy	151	25.2	0.63	0.47	0.86
Robert C et al. (Robert et al., 2019)	2019	NCT01866319	KEYNOTE-006	834	3	Melanoma	Pembrolizumab	556	Ipilimumab	278	57.7	0.73	0.61	0.88
Kojima T et al. (Kojima et al., 2020)	2020	NCT02564263	KEYNOTE-181	628	3	Esophageal cancer	Pembrolizumab	314	Chemotherapy	314	7.1	0.89	0.75	1.05
Popat S et al. (Popat et al., 2020)	2020	NCT02991482	ETOP 9-15	144	3	Malignant pleural mesothelioma	Pembrolizumab	73	Chemotherapy	71	17.5	1.04	0.66	1.67
Shitara K et al. (Shitara et al., 2020)	2020	NCT02494583	KEYNOTE-062	506	3	Gastric/gastrooesophageal junction cancer	Pembrolizumab	256	Chemotherapy	250	29.4	0.91	0.69	1.18
Powles T et al. (Powles et al., 2021)	2021	NCT02853305	KEYNOTE-361	659	3	Urothelial carcinoma	Pembrolizumab	307	Chemotherapy	352	31.7	0.92	0.77	1.11
Winer EP et al. (Winer et al., 2021)	2021	NCT02555657	KEYNOTE-119	622	3	Breast cancer	Pembrolizumab	312	Chemotherapy	310	31.4	0.97	0.82	1.15

OS = Overall survival. HR = Hazard ratio. CI = Confidence interval.

**TABLE 3:** List of the studies involving serious AEs in this meta-analysis.

First author	Year	NCT number	Trail name	Total number	Cancer type	Trial phase	Treatment	Patient number	Total number surveyed	Grade 3 or higher AEs
Fehrenbacher L et al. (Fehrenbacher et al., 2016)	2016	NCT01903993	POPLAR	287	Non small cell lung cancer	2	Atezolizumab	144	142	17
						2	Standard	143	135	55
Fehrenbacher L et al. (Fehrenbacher et al., 2018)	2018	NCT02008227	OAK	1225	Non small cell lung cancer	3	Atezolizumab	613	609	91
						3	Standard	612	578	246
McDermott DF et al. (McDermott et al., 2018)	2018	NCT01984242	IMmotion150	204	Renal cell carcinoma	2	Atezolizumab	103	103	17
						2	Standard	101	100	57
Powles T et al. (Powles et al., 2018)	2018	NCT02302807	IMvigor211	234	Urothelial carcinoma	3	Atezolizumab	116	114	11
						3	Standard	118	112	43
Eng C et al. (Eng et al., 2019)	2019	NCT02788279	IMblaze370	180	Colorectal cancer	3	Atezolizumab	90	90	28
						3	Standard	90	80	46
Herbst RS et al. (Herbst et al., 2020)	2020	NCT02409342	IMpower110	554	Non small cell lung cancer	3	Atezolizumab	277	286	97
						3	Standard	277	263	149
Bang YJ et al. (Bang et al., 2018)	2018	NCT02625623	JAVELIN Gastric 300	371	Gastric/gastrooesophageal junction cancer	3	Avelumab	185	184	17
						3	Standard	186	177	56
Park K et al. (Park et al., 2021)	2021	NCT02395172	JAVELIN Lung 200	529	Non small cell lung cancer	3	Avelumab	264	393	41
						3	Standard	265	365	180
Pujade-Lauraine E et al. (Pujade-Lauraine et al., 2021)	2021	NCT02580058	JAVELIN Ovarian 200	378	Ovarian cancer	3	Avelumab	188	187	30
						3	Standard	190	177	56
Huang J et al. (Huang et al., 2020)	2020	NCT03099382	ESCORT	448	Squamous cell carcinoma	3	Camrelizumab	228	228	44
						3	Standard	220	220	87
Sezer A et al. (Sezer et al., 2021)	2021	NCT03088540	EMPOWER-Lung 1	563	Non small cell lung cancer	3	Cemiplimab	283	355	50
						3	Standard	280	342	134
O'Reilly EM et al. (O'Reilly et al., 2019)	2019	NCT02558894	NA	65	Pancreatic ductal adenocarcinoma	2	Durvalumab+Tremelimumab	32	32	7
						2	Durvalumab	33	32	2
Siu LL et al. (Siu et al., 2019)	2019	NCT02319044	CONDOR	267	Squamous Cell Carcinoma	2	Durvalumab+Tremelimumab	133	133	21
						2	Durvalumab	67	65	8
						2	Tremelimumab	67	65	11
Ferris RL et al. (Ferris et al., 2020)	2020	NCT02369874	EAGLE	736	Squamous cell carcinoma	3	Durvalumab	240	237	24
						3	Durvalumab+Tremelimumab	247	246	40
						3	Standard	249	240	58
Planchard D et al. (Planchard et al., 2020)	2020	NCT02352948	ARCTIC	595	Non small cell lung cancer	3	Durvalumab	62	62	6
						3	Durvalumab+Tremelimumab	174	173	38
						3	Durvalumab	117	117	14
						3	Tremelimumab	60	60	14
						3	Standard	64	63	28
						3	Standard	118	110	40
Powles T et al. (Powles et al., 2020)	2020	NCT02516241	DANUBE	1032	Urothelial carcinoma	3	Durvalumab	346	345	49
						3	Durvalumab+Tremelimumab	342	340	95
						3	Standard	344	313	189
Rizvi NA et al. (Rizvi et al., 2020)	2020	NCT02453282	MYSTIC	488	Non small cell lung cancer	3	Durvalumab	163	369	55
						3	Durvalumab+Tremelimumab	163	371	85
						3	Standard	162	352	119
Bachelot T et al. (Bachelot et al., 2021)	2021	NCT02299999	SAFIR02-BREAST IMMUNO	199	Breast cancer	2	Durvalumab	68	63	10
						2	Standard	131	129	17
Hodi FS et al. (Hodi et al., 2010)	2010	NCT00094653	MDX010-20	273	Melanoma	3	Ipilimumab	137	131	30
						3	Standard	136	132	15
Bang YJ et al. (Bang et al., 2017)	2017	NCT01585987	NA	108	Gastric/gastrooesophageal junction cancer	2	Ipilimumab	57	57	13
						2	Standard	51	45	4
Borghaei H et al. (Borghaei et al., 2015)	2015	NCT01673867	CheckMate 057	582	Non small cell lung cancer	3	Nivolumab	292	287	30
						3	Standard	290	268	144
Brahmer J et al. (Brahmer et al., 2015)	2015	NCT01642004	CheckMate 017	272	Non small cell lung cancer	3	Nivolumab	135	131	9
						3	Standard	137	129	71
Motzer RJ et al. (Motzer et al., 2015)	2015	NCT01668784	CheckMate 025	821	Renal cell carcinoma	3	Nivolumab	410	406	76
						3	Standard	411	397	145
Ferris RL et al. (Ferris et al., 2016)	2016	NCT02105636	CheckMate 141	361	Squamous cell carcinoma	3	Nivolumab	240	236	31
						3	Standard	121	111	39
Hodi FS et al. (Hodi et al., 2016)	2016	NCT01927419	CheckMate 069	142	Melanoma	2	Nivolumab+Ipilimumab	95	94	51
						2	Ipilimumab	47	46	9
Carbone DP et al. (Carbone et al., 2017)	2017	NCT02041533	CheckMate 026	541	Non small cell lung cancer	3	Nivolumab	271	267	47
						3	Standard	270	263	133
Weber J et al. (Weber et al., 2017)	2017	NCT02388906	CheckMate 238	906	Melanoma	3	Nivolumab	453	452	65
						3	Ipilimumab	453	453	208
Amaria RN et al. (Amaria et al., 2018)	2018	NCT02519322	NA	23	Melanoma	2	Nivolumab	12	12	1
						2	Nivolumab+Ipilimumab	11	11	8
Hodi FS et al. (Hodi et al., 2018)	2018	NCT01844505	CheckMate 067	945	Melanoma	3	Nivolumab+Ipilimumab	314	313	185
						3	Ipilimumab	315	311	86
						3	Nivolumab	316	313	70
Larkin J et al. (Larkin et al., 2018)	2018	NCT01721746	CheckMate 037	405	Melanoma	3	Nivolumab	272	268	37
						3	Standard	133	102	84
Ascierto PA et al. (Ascierto et al., 2019)	2019	NCT01721772	CheckMate 066	418	Melanoma	3	Nivolumab	210	206	31
						3	Standard	208	205	36
Hellmann MD et al. (Hellmann et al., 2019)	2019	NCT02477826	CheckMate 227	1166	Non small cell lung cancer	3	Nivolumab+Ipilimumab	583	576	189
						3	Standard	583	570	205
Kato K et al. (Kato et al., 2019)	2019	NCT02569242	ATTRACTION-3	419	Squamous cell carcinoma	3	Nivolumab	210	209	38
						3	Standard	209	208	133
Scherpereel A et al. (Scherpereel et al., 2019)	2019	NCT02716272	IFCT-1501 MAPS2	125	Malignant pleural mesothelioma	2	Nivolumab	63	63	9
						2	Nivolumab+Ipilimumab	62	61	16
Wu YL et al. (Wu et al., 2019)	2019	NCT02613507	CheckMate 078	504	Non small cell lung cancer	3	Nivolumab	338	337	35
						3	Standard	166	156	74
Motzer RJ et al. (Motzer et al., 2020)	2020	NCT02231749	CheckMate 214	1096	Renal cell carcinoma	3	Nivolumab+Ipilimumab	550	547	259
						3	Standard	546	535	343
Reardon DA et al. (Reardon et al., 2020)	2020	NCT02017717	CheckMate 143	369	Glioblastoma	3	Nivolumab	184	182	33
						3	Standard	185	165	25
Zimmer L et al. (Zimmer et al., 2020)	2020	NCT02523313	IMMUNED	115	Melanoma	2	Nivolumab+Ipilimumab	56	55	39
						2	Nivolumab	59	56	15
Baas P et al. (Baas et al., 2021)	2021	NCT02899299	CheckMate 743	605	Malignant pleural mesothelioma	3	Nivolumab+Ipilimumab	303	300	91
						3	Standard	302	284	91
Owonikoko TK et al. (Owonikoko et al., 2021)	2021	NCT02538666	CheckMate 451	559	Small cell lung cancer	3	Nivolumab+Ipilimumab	279	278	145
						3	Nivolumab	280	279	32
Spigel DR et al. (Spigel et al., 2021)	2021	NCT02481830	CheckMate 331	569	Small cell lung cancer	3	Nivolumab	284	282	39
						3	Standard	285	265	194
Tannir NM et al. (Tannir et al., 2021)	2021	NA	CheckMate 214	139	Renal cell carcinoma	3	Nivolumab+Ipilimumab	74	73	36
						3	Standard	65	65	29
Ribas A et al. (Ribas et al., 2013)	2013	NCT00257205	NA	655	Melanoma	3	Tremelimumab	328	325	192
						3	Standard	327	319	132
Herbst RS et al. (Herbst et al., 2016)	2016	NCT01905657	KEYNOTE-010	687	Non small cell lung cancer	2/3	Pembrolizumab	344	339	43
						2/3	Standard	343	309	109
Hamid O et al. (Hamid et al., 2017)	2017	NCT01704287	KEYNOTE-002	359	Melanoma	2	Pembrolizumab	180	178	24
						2	Standard	179	171	45
Shitara K et al. (Shitara et al., 2018)	2018	NCT02370498	KEYNOTE-061	395	Gastric/gastrooesophageal junction cancer	3	Pembrolizumab	196	294	42
						3	Standard	199	276	96
Cohen EEW et al. (Cohen et al., 2019)	2019	NCT02252042	KEYNOTE-040	495	Squamous cell carcinoma	3	Pembrolizumab	247	246	33
						3	Standard	248	234	85
Fradet Y et al. (Fradet et al., 2019)	2019	NCT02256436	KEYNOTE-045	542	Urothelial cancer	3	Pembrolizumab	270	266	44
						3	Standard	272	255	128
Mok TSK et al. (Mok et al., 2019)	2019	NCT02220894	KEYNOTE-042	1274	Non-small cell lung cancer	3	Pembrolizumab	637	636	113
						3	Standard	637	615	252
Reck M et al. (Reck et al., 2019)	2019	NCT02142738	KEYNOTE-024	305	Non-small cell lung cancer	3	Pembrolizumab	154	154	48
						3	Standard	151	150	80
Robert C et al. (Robert et al., 2019)	2019	NCT01866319	KEYNOTE-006	834	Melanoma	3	Pembrolizumab	556	555	103
						3	Ipilimumab	278	256	54
André T et al. (André et al., 2020)	2020	NCT02563002	KEYNOTE-177	307	Colorectal cancer	3	Pembrolizumab	153	153	86
						3	Standard	154	143	111
Kojima T et al. (Kojima et al., 2020)	2020	NCT02564263	KEYNOTE-181	628	Esophageal cancer	3	Pembrolizumab	314	314	57
						3	Standard	314	296	121
Popat S et al. (Popat et al., 2020)	2020	NCT02991482	ETOP 9-15	144	Malignant pleural mesothelioma	3	Pembrolizumab	73	72	14
						3	Standard	71	70	18
Kuruvilla J et al. (Kuruvilla et al., 2021)	2021	NCT02684292	KEYNOTE-204	304	Hodgkin lymphoma	3	Pembrolizumab	151	148	29
						3	Standard	153	152	38

AEs = Adverse events.

PFS is considered to be the primary endpoint of randomized clinical trials evaluating patients with solid tumors (Korn and Crowley, 2013). OS is defined as the time from the start of treatment to death or the last follow-up. The HRs of PFS and OS represent HRs between treatment 1 and 2. In assessing AEs, we chose treatment-related or drug-related AEs as the main results. If there were no treatment or drug-related AEs in studies, we included all AEs. The classification of AEs is often used to evaluate the type and severity of AEs in clinical trials. According to AE classification, grade 3 or higher AEs are considered as severe AEs. The risk of severe AEs is the focus of the evaluation of therapeutic effectiveness, so the number of AEs surveyed and severe AEs were both extracted (Xu et al., 2018).

### 2.3 Data Synthesis and Statistical Analysis

#### 2.3.1 Adverse Events Analysis

We used gemtc and pcnetmeta packages in R v4.0.3 and called JAGS v4.3.0 to perform statistical analysis in a Bayesian framework based on Markov Chain Monte Carlo (MCMC) methods, and generated the graph depicting the network geometry (Wang et al., 2019).

Firstly, we made a rough comparison between the fit of the consistent model with the inconsistent model. Secondly, the inconsistency on the specific comparison was tested by node splitting analysis. *p* < 0.05 was considered as indicating a significant inconsistency. Outstanding consistency is the key to robust results, as evidenced by the consistency between direct and indirect results. We compared the results of network meta-analysis (indirect results) with those of pairwise analysis (direct results) to explore the sources of inconsistency. Additionally, if there existed significant heterogeneity, we used the random-effect model. Otherwise, we used the fixed-effect model (Dias et al., 2011). We used non-information prior distributions and overdispersed initial values (scaling 2.5) in 3 chains to fit the model. 56 independent randomized controlled experiments yielded 100,000 iterations (including 20,000 optimization iterations) with 10 refinement intervals for each chain. This method was used to generate a posterior distribution of model parameters. The convergence of iterations was evaluated by using the Gelman-Rubin-Brooks statistics, all of which converge near 1. Based on the odds ratio (OR) advantage ratio and posterior probability, we ranked probabilities of each treatment as the safest, followed by the second, third and so on.

### 2.4 Progression-free Survival and Overall Survival Analysis

For the consistency and heterogeneity analysis of PFS and OS, we chose to use R’s netmeta package in the Frequentist framework to make a preliminary judgment using the traditional frequency method, avoiding the artificial bias caused by complex prior settings, settings of dummy variables and variance-covariance matrices of regression models in Bayesian statistics, which would simplify the operator’s parameter setting. The I^2^ test was used to evaluate the heterogeneity between studies, with the significance level set as *p* < 0.05. I^2^ greater than 25, 50 or 75% indicated low, medium and high heterogeneity respectively. If significant heterogeneity exists, the random-effect model was used. Otherwise, we employed the fixed-effect model (Higgins and Thompson, 2002).

Since Bayesian statistics are more accurate and the results are highly consistent with those in the frequency model, we subsequently chose the Bayesian framework by using the MCMC method in WinBUGS v1.4.3 for network meta-analysis. We used the consistency model (due to I^2^ < 25) to calculate HRs and 95% CIs. We simulated 3 different chains, each with 45,000 built-in samples, resulting in 15 iterations with a refinement rate of 15 (3 different chains with 15,000 iterations and 45,000 burn-in samples and 50 thinning rates). The model fitting was further determined according to the deviation information criterion. The output was a posterior distribution of relative effect size, and we got the estimated average of HR and 95% CI (95% CI as the 2.5th and 97.5th percentiles) (van Valkenhoef et al., 2012). The ranking probability distribution was calculated, ranking the probabilities of each treatment as the safest, followed by the second, third and so on.

## 3 Results

### 3.1 Literature Search and Study Characteristics

**FIGURE 1:**
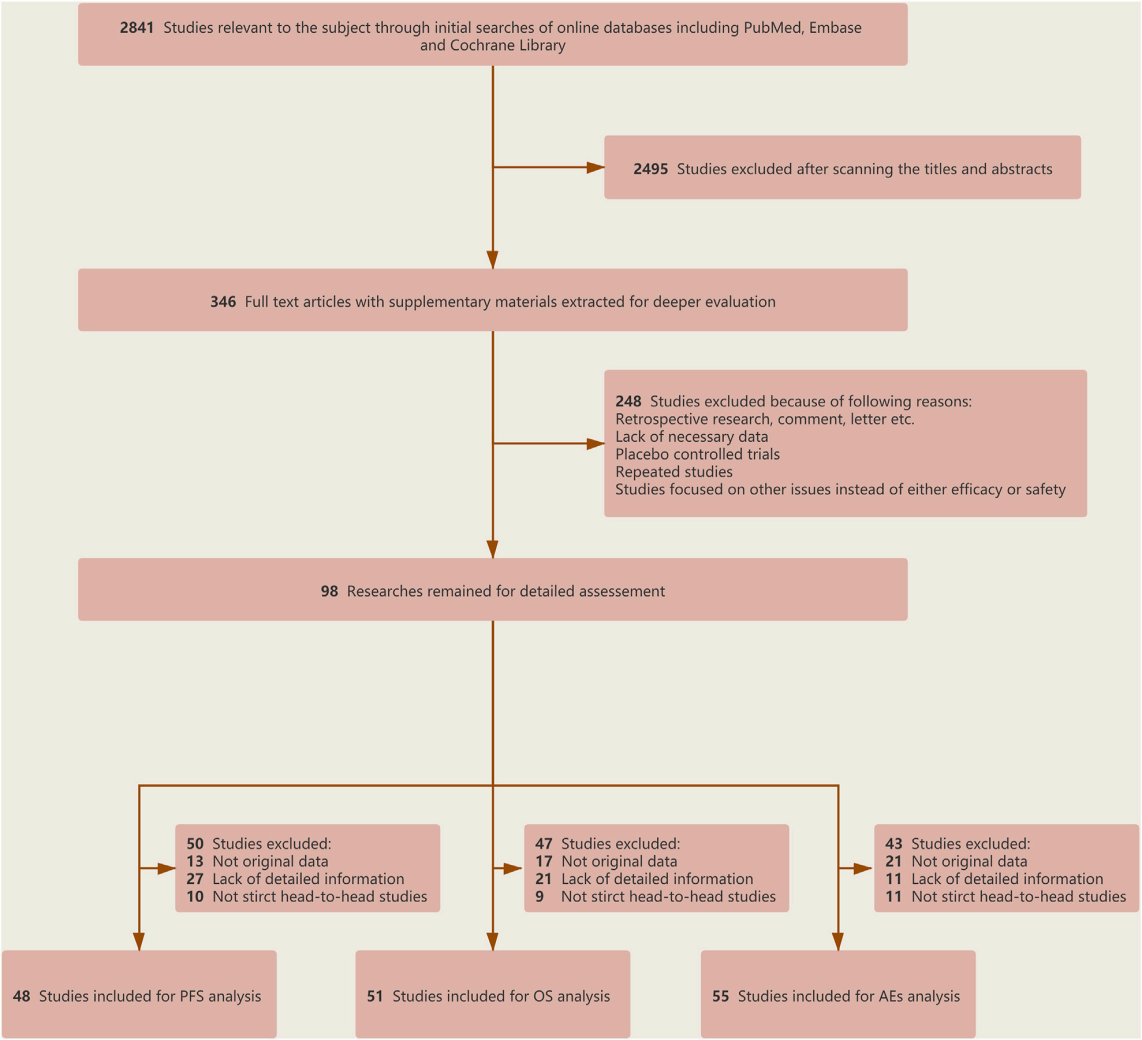
Flowchart of selection criteria and study design.

**FIGURE 2:**
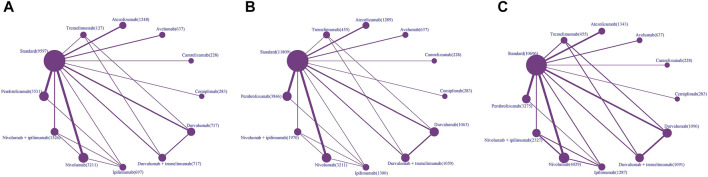
Network plots of comparisons for PFS **(A)**, OS **(B)** and AEs **(C)** of different types of treatment-based network meta-analysis. Each node represents a treatment. The size of the circle is in proportion to the number of patients. The line width is in proportion to the number of patients included in the direct comparison of two treatments.

After a preliminary search, a total of 2,841 related articles were identified. After the screening of the title and abstract, 2,495 studies were excluded because they did not meet the corresponding standards. We carefully reviewed the remaining studies and then incorporated 63 RCTs for final analysis (2,14–75). The literature selection flowchart is shown in Figure 1. Of these, 48 RCTs involving 22,519 patients were analyzed for HRs of PFS, 51 RCTs involving 27,150 patients were analyzed for HRs of OS, and 55 RCTs involving 26,747 patients were analyzed for severe AEs (Figure 2).

In terms of PFS, ICI drugs included nivolumab (*n* = 13), pembrolizumab (*n* = 13), atezolizumab (*n* = 6), durvalumab (*n* = 6), ipilimumab (*n* = 5), avelumab (*n* = 3), tremelimumab (*n* = 2), camrelizumab (*n* = 1), cemiplimab (*n* = 1), nivolumab plus ipilimumab (*n* = 7), durvalumab plus tremelimumab (*n* = 5). Cancer types tested in these studies include lung cancer (*n* = 16), melanoma (*n* = 6), squamous cell carcinoma (*n* = 6), gastric/gastrooesophageal junction cancer (*n* = 4), renal cell carcinoma (*n* = 4), colorectal cancer (*n* = 2), malignant pleural mesothelioma (*n* = 2), ovarian cancer (*n* = 2), urothelial cancer (*n* = 2), breast cancer (*n* = 1), esophageal cancer (*n* = 1), glioblastoma (*n* = 1), Hodgkin lymphoma (*n* = 1) (Figure 2A).

In terms of OS, ICI drugs included nivolumab (*n* = 13), pembrolizumab (*n* = 13), durvalumab (*n* = 7), atezolizumab (*n* = 6), ipilimumab (*n* = 6), avelumab (*n* = 3), tremelimumab (*n* = 3), camrelizumab (*n* = 1), cemiplimab (*n* = 1), nivolumab plus ipilimumab (*n* = 7), durvalumab plus tremelimumab (*n* = 6). Cancer types tested in these studies include lung cancer (*n* = 17), melanoma (*n* = 9), squamous cell carcinoma (*n* = 6), urothelial cancer (*n* = 4), gastric/gastrooesophageal junction cancer (*n* = 3), renal cell carcinoma (*n* = 3), breast cancer (*n* = 2), colorectal cancer (*n* = 1), malignant pleural mesothelioma (*n* = 2), ovarian cancer (*n* = 2), esophageal cancer (*n* = 1), glioblastoma (*n* = 1) (Figure 2B).

In terms of severe AEs, ICI drugs included nivolumab (*n* = 17), pembrolizumab (*n* = 12), durvalumab (*n* = 8), atezolizumab (*n* = 6), ipilimumab (*n* = 6), avelumab (*n* = 3), tremelimumab (*n* = 3), camrelizumab (*n* = 1), cemiplimab (*n* = 1), nivolumab plus ipilimumab (*n* = 11), durvalumab plus tremelimumab (*n* = 7). Cancer types tested in these studies include lung cancer (*n* = 17), melanoma (*n* = 11), squamous cell carcinoma (*n* = 6), renal cell carcinoma (*n* = 4), gastric/gastrooesophageal junction cancer (*n* = 3), malignant pleural mesothelioma (*n* = 3), urothelial cancer (*n* = 3), colorectal cancer (*n* = 2), breast cancer (*n* = 1), esophageal cancer (*n* = 1), glioblastoma (*n* = 1), hodgkin lymphoma (*n* = 1), ovarian cancer (*n* = 1), pancreatic ductal adenocarcinoma (*n* = 1) (Figure 2C).

### 3.2 Progression-free Survival

**FIGURE 3:**
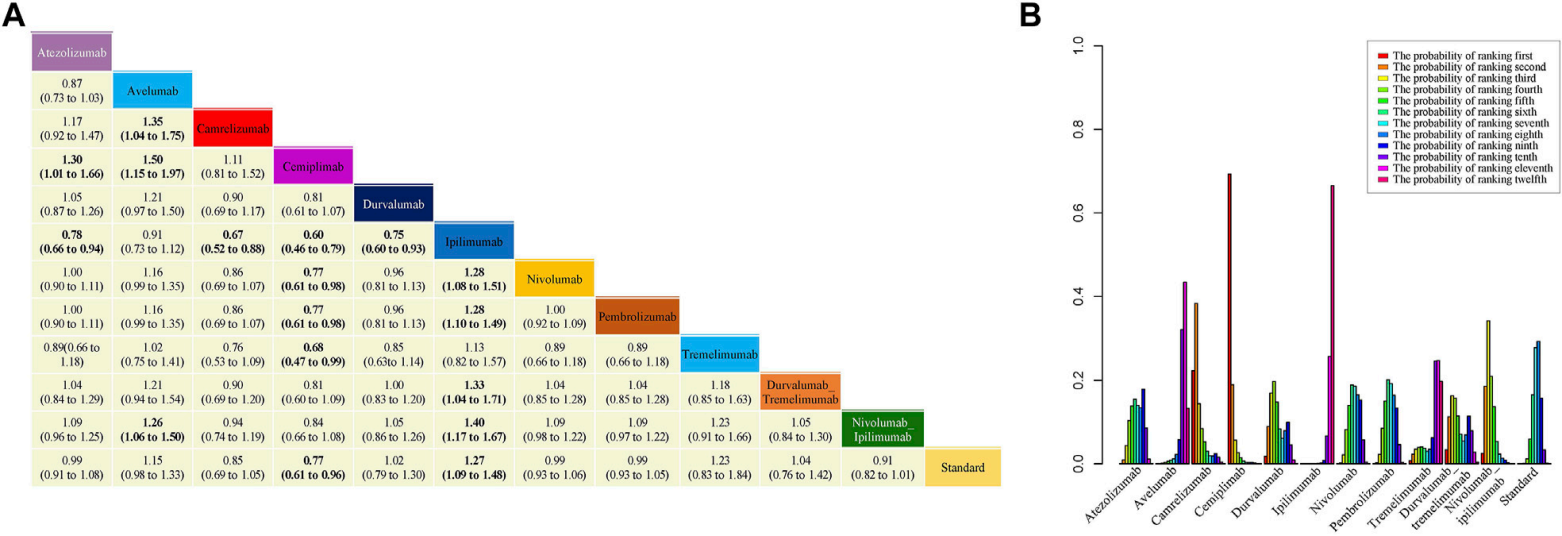
Results of network meta-analysis (NMA), safety profile **(A)** and probability ranking diagram **(B)** in the Bayesian model. In the safety profile, efficacy of treatment for progression-free survival (PFS) is represented as hazard ratios (HRs) with 95% confidence intervals. All comparisons are made as column versus row. Statistically significant results are in bold. Probability ranking diagram shows the probability of the safety of different therapies ranking the first to the last for PFS.

In analyzing PFS, no significant heterogeneity (I^2^ = 19%) or inconsistency was observed (*p* = 0.97) (Supplementary Table S2). Therefore, the Bayesian fixed-effect model was used. HRs and 95% CI from the network meta-analysis are shown in Figure 3A. Treatment with ipilimumab was significantly worse in terms of PFS than standard therapies, whereas treatment with cemiplimab significantly improved PFS. According to the probability ranking diagram, the results showed that cemiplimab was the best choice in terms of PFS, camrelizumab ranked the second safest and nivolumab plus ipilimumab ranked the third safest, whereas ipilimumab was the worst (Figure 3B). Additionally, treatment with ipilimumab was significantly worse than most other treatments in terms of PFS. Interestingly, nivolumab plus ipilimumab significantly improved PFS compared to ipilimumab, which suggested that treatment with combinations of ICI drugs may benefit PFS compared to monotherapy. The results calculated according to the frequency model were highly consistent with the results of the Bayesian fixed-effect model.

Additionally, we performed subgroup analyses based on treatment of different cancer types, particularly lung cancer and melanoma. The safety profile and probability ranking diagram for lung cancer and melanoma are shown in Supplementary Figures S1, S4 in the Supplement respectively. Cemiplimab was also the best choice in terms of PFS in treating lung cancer, and nivolumab plus ipilimumab ranked the second safest. Compared with standard therapies, HR (95% CI) for cemiplimab was 0.77 (0.61–0.96). Treatment with cemiplimab also significantly improved PFS compared to nivolumab. Tremelimumab was considered the worst choice in terms of PFS in treating lung cancer. In terms of melanoma, our results showed that nivolumab plus ipilimumab was the best choice for PFS. HR (95% CI) for nivolumab plus ipilimumab was 0.69 (0.52–0.92) compared with standard therapies. In addition, HRs (95% CI) for nivolumab and pembrolizumab were 0.78 (0.65–0.94) and 0.80 (0.64–0.99) respectively. The probability ranking diagram of melanoma indicated that ipilimumab was the worst choice for PFS.

### 3.3 Overall Survival

**FIGURE 4:**
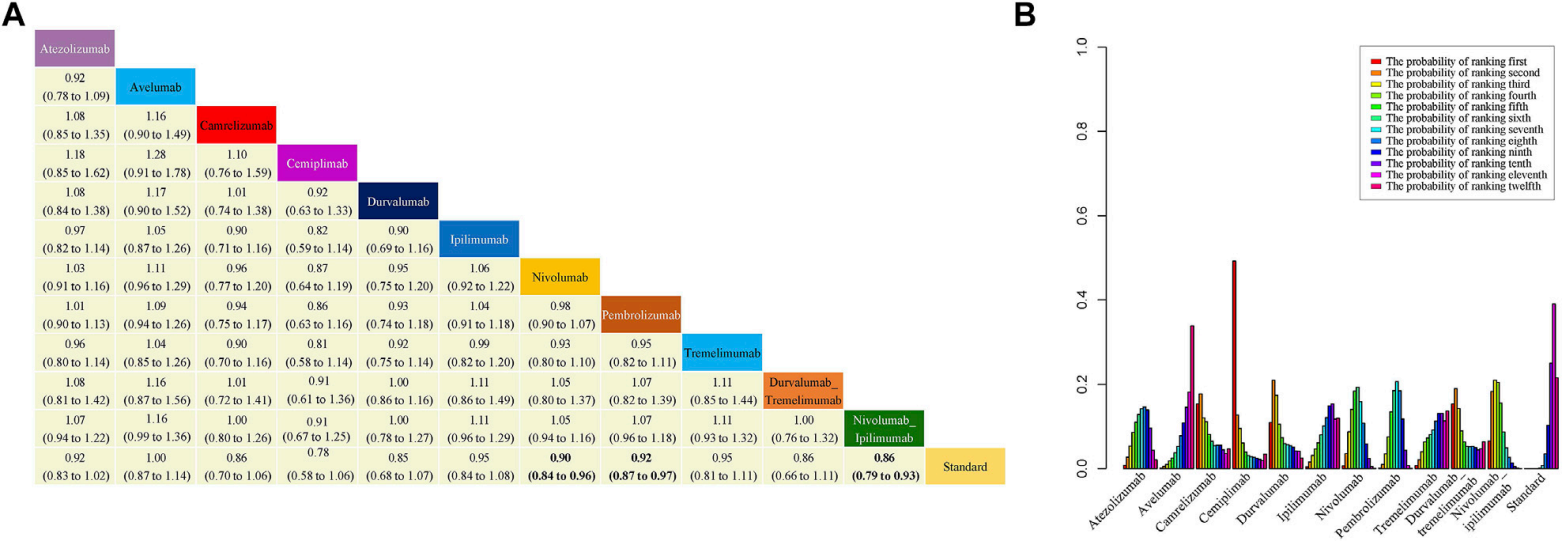
Results of NMA, safety profile **(A)** and probability ranking diagram **(B)**. In the safety profile, efficacy of treatment for overall survival (OS) is represented as HRs with 95% confidence intervals. All comparisons are made as column versus row. Statistically significant results are in bold. Probability ranking diagram shows the probability of the safety of different therapies ranking the first to the last for OS.

In analyzing OS, no consistency (I^2^ = 0%) or inconsistency (*p* = 0.60) (Supplementary Table S2) was observed, and so the Bayesian fixed-effects model was applied. HRs and 95% CI are shown in Figure 4A. Treatment with nivolumab, pembrolizumab and nivolumab plus ipilimumab significantly improved OS compared to standard therapies. According to the probability ranking diagram, cemiplimab was the best choice in terms of OS, and durvalumab ranked the second safest, whereas avelumab was the worst (Figure 4B). Of note, nivolumab plus ipilimumab may improve OS compared with nivolumab and ipilimumab monotherapy, which was similar to durvalumab plus tremelimumab compared with durvalumab and tremelimumab monotherapy. The results calculated based on the frequency model were also highly similar to the results of the Bayesian fixed-effect model.

We also conducted subgroup analyses for lung cancer and melanoma. The safety profiles and probability ranking diagrams of lung cancer and melanoma are shown in Supplementary Figures S5, S8 in the Supplement. For lung cancer treatment, cemiplimab was the best option for OS and durvalumab ranked the second safest. Safety profile of lung cancer suggested that compared with standard therapies, HRs (95% CI) for nivolumab, nivolumab plus ipilimumab and pembrolizumab were 0.91 (0.82–0.99), 0.87 (0.76–0.99), and 0.89 (0.80–0.99) respectively. Standard therapies were considered to be the worst option for lung cancer in terms of OS whereas. For melanoma treatment, nivolumab plus ipilimumab was the best option. Safety profiles showed that HR (95% CI) for nivolumab was 0.84 (0.71–0.99) compared with standard therapies. Our results also indicated that standard therapies were the worst choice for melanoma in terms of OS.

### 3.4 Severe Adverse Events

**FIGURE 5:**
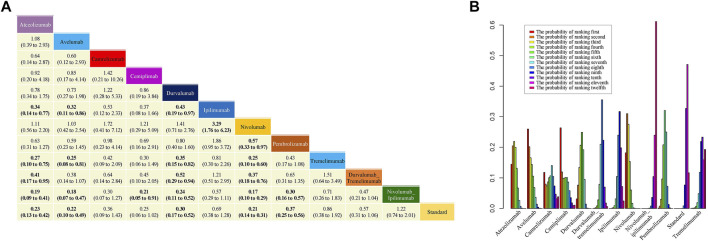
Results of NMA, safety profile **(A)** and probability ranking diagram **(B)**. In the safety profile, efficacy of treatment for grade 3–5 adverse events is represented as odd ratios (ORs) with 95% confidence intervals. All comparisons are made as column versus row. Statistically significant results are in bold. Probability ranking diagram shows the probability of the safety of different therapies ranking the first to the last for severe adverse events.

In the network meta-analyses of severe AEs, high heterogeneity was found (Supplememntary Table S3), and the random-effect model was employed. Safety profile in the consistency model is shown in Figure 5A. Atezolizumab, avelumab, durvalumab, nivolumab, and pembrolizumab all significantly reduced the risk of grade 3 or higher AEs compared to standard therapies. Compared with standard therapies, ORs (95% CI) for atezolizumab, avelumab, durvalumab, nivolumab, and pembrolizumab were 0.23 (0.13–0.42), 0.22 (0.10–0.49), 0.30 (0.17–0.52), 0.21 (0.14–0.31), and 0.37 (0.25–0.56) respectively. It is worth noting that there was no direct evidence that durvalumab plus tremelimumab could reduce the risk of severe AEs compared to durvalumab and tremelimumab monotherapy. Similarly, there was no evidence that the combination of nivolumab and ipilimumab could significantly reduce the risk of AEs compared with nivolumab and ipilimumab monotherapy. Even combination therapies increased the risk of severe AEs (durvalumab vs. durvalumab plus tremelimumab: [OR], 0.52%; 95% CI, 0.29–0.94; nivolumab vs. nivolumab plus ipilimumab: [OR], 0.17%; 95% CI, 0.10–0.29).

Figure 5B shows the probability ranking diagram of 12 interventions. The probabilities of becoming the safest choice for severe AEs were as follows: cemiplimab (26.3%), avelumab (25.9%), nivolumab (18.2%), atezolizumab (14.5%), and camrelizumab (11.8%). The remaining interventions were all less than 5% likely to be the safest option, with nivolumab plus ipilimumab appearing to be the worst choice.

Through node splitting analysis, significant inconsistency could not be detected for most comparisons (Supplementary Table S4). There was significant inconsistency between ipilimumab and nivolumab plus ipilimumab, and ipilimumab and standard therapies (*p* < 0.05). The comparison between nivolumab plus ipilimumab and standard therapies also showed a degree of inconsistency (*p* = 0.08). In the direct comparisons, patients receiving nivolumab plus ipilimumab were more likely to have severe AEs than those receiving ipilimumab, and patients receiving ipilimumab were more likely to have severe AEs than those receiving standard therapies. However, patients receiving standard therapies were more likely to have severe AEs than those receiving nivolumab plus ipilimumab. The comparison between the above three groups may be the main reason for the inconsistency.

We performed subgroup analyses of lung cancer, melanoma, and squamous cell carcinoma treatment. The respective safety profiles and probability ranking diagram are shown in Supplementary Figures S9, S14 in the Supplement. Interestingly, the results suggested that nivolumab was the joint best choice for lung cancer, melanoma and squamous cell carcinoma. Standard therapies, based on the probability ranking diagram, were considered to be the worst for lung cancer and squamous cell carcinoma treatment, and nivolumab plus ipilimumab was the worst for melanoma treatment.

## 4 Discussion

In order to comprehensively evaluate the safety and efficacy of ICI drug monotherapy and combination therapies, we conducted a network meta-analysis combining HRs of PFS and OS, and the risk of severe AEs, and performed subgroup analyses particularly for lung cancer and melanoma. The application of bioinformatics is often used to analyze published data (Wu et al., 2017). To our knowledge, this study is the first comprehensive report comparing PFS HRs, OS HRs, and corresponding treatment-related severe AEs among ICI drug monotherapy, combination therapies and standard therapies.

In terms of PFS and OS, we first tested the heterogeneity and consistency of network meta-analysis based on the frequency method. No significant heterogeneity and consistency were found, indicating that this network meta-analysis was consistent in PFS and OS. We used the frequency model and the Bayesian model separately. The results of the frequency model and the Bayesian model agree well. In view of the greater accuracy of the Bayesian model, our final results were presented by the Bayesian model.

In terms of PFS, treatment with ipilimumab was significantly worse than standard therapies, whereas treatment with cemiplimab significantly improved PFS. The results also indicated that cemiplimab was the best choice for PFS. Treatment with nivolumab, pembrolizumab and nivolumab plus ipilimumab significantly improved OS compared to standard therapies. For OS, cemiplimab was considered to be the best choice, whereas avelumab was the worst. Since few studies compared cemiplimab and camrelizumab with other therapies, nivolumab plus ipilimumab ranked the third safest in PFS and durvalumab ranked the second safest in terms of OS. In comparing ICI drug combination therapies with monotherapy, we found that nivolumab plus ipilimumab significantly improved PFS compared to ipilimumab. Additionally, nivolumab plus ipilimumab may improve OS compared with nivolumab and ipilimumab monotherapy, similar to durvalumab plus tremelimumab compared with durvalumab, and tremelimumab monotherapy. However, further studies are needed to compare the safety and efficacy of ICI drug combination treatment with monotherapy. Although abundant included studies may lead to low significance of the overall results in terms of PFS and OS, we thought that the results would be more reliable and further performed subgroup analysis.

Severe AEs were examined as the measure of the toxicity of different ICI drug therapies and standard therapies. In terms of severe AEs, there was a large inconsistency in the comparison between the above three groups, which is similar to inconsistency reported by Chen *et al* (Xu et al., 2018), but the degree of inconsistency was more obvious in the comparison between ipilimumab and standard therapies in this study. We considered the main reasons as follows: In spite that the inclusion and exclusion criteria in our study are similar to those of Chen *et al*, eligible studies with inconsistent results were relatively abundant, which may lead to higher inconsistency. Of note, Chen *et al* combined durvalumab plus tremelimumab and nivolumab plus ipilimumab as the two ICI drug group, however, we grouped them in our study to assess the safety and efficacy more precisely.

### 4.1 Strengths and Limitations

The main strengths of our studies are as follows: we used the Bayesian model to conduct network meta-analysis and then employed the frequency model for inconsistency test and result verification in terms of PFS and OS. We found that the results of the frequency model were highly consistent with those of the Bayesian model, and we represented our final results from the Bayesian model, which greatly enhanced the reliability of conclusions. To comprehensively investigate the safety and efficacy of various ICI drugs, we analyzed three different indicators PFS, OS and severe AEs. Of note, we included enough studies to ensure the accuracy of the results. Despite that some studies represent the data of the same RCTs at different times, we chose the most recent results as much as possible.

This study also has several limitations. Firstly, enrolled studies showed high heterogeneity. In order to avoid publication bias, we tested the heterogeneity and used different models accordingly. Secondly, the number of RCTs that meet the requirements for inclusion is different among ICI drugs at present, and there is obvious inconsistency in severe AEs, which require more studies for higher-level verification. Thirdly, despite randomization of the eligible studies, there are still characteristic imbalances between the groups in trials.

## 5 Conclusion

In the present study, the Bayesian model was used to comprehensively assess survival data and the risk of severe AEs for ICI drugs, which showed that different ICI drug therapies may pose different risks in terms of PFS, OS and severe AEs. Our study may provide new insights and strategies for the clinical practice of ICI drugs.

## Data Availability

The raw data supporting the conclusion of this article will be made available by the authors, without undue reservation.
