# Supplementary material for: Comprehensive Evaluation of Anti-PD-1, Anti-PD-L1, Anti-CTLA-4 and Their Combined Immunotherapy in Clinical Trials: A Systematic Review and Meta-analysis
Source: Front Pharmacol. 2022 May 25;13:883655. doi: 10.3389/fphar.2022.883655 (PMC9174611; doi:10.3389/fphar.2022.883655)
Supplement: Supplementary file 1 [file DataSheet1.docx]

**
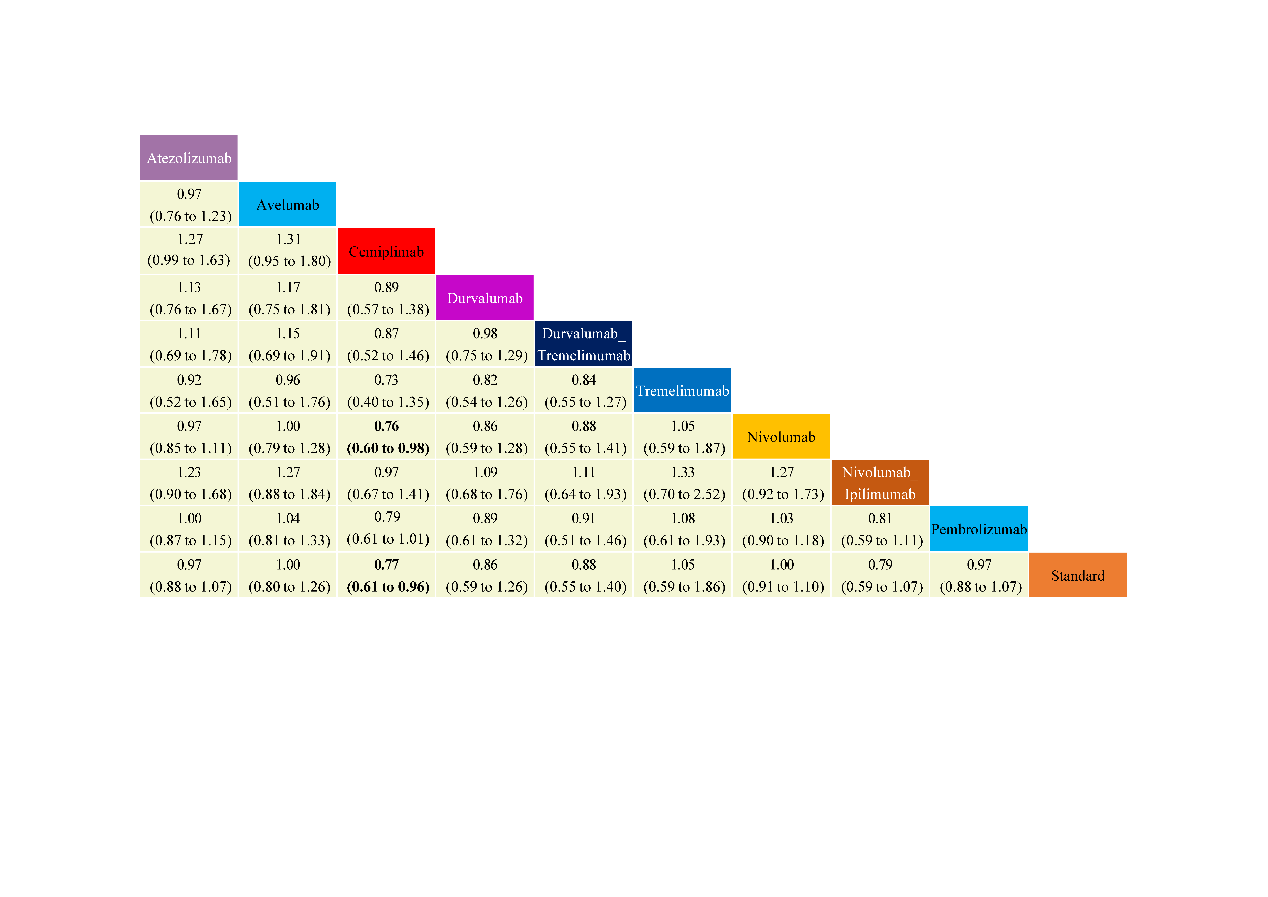
**

**Figure S1 Safety profile in the lung cancer subgroup for PFS.** In the safety profile, efficacy of treatment for PFS is represented as HRs with 95% credibility intervals. All comparisons are made as column versus row. Statistically important results are in bold.


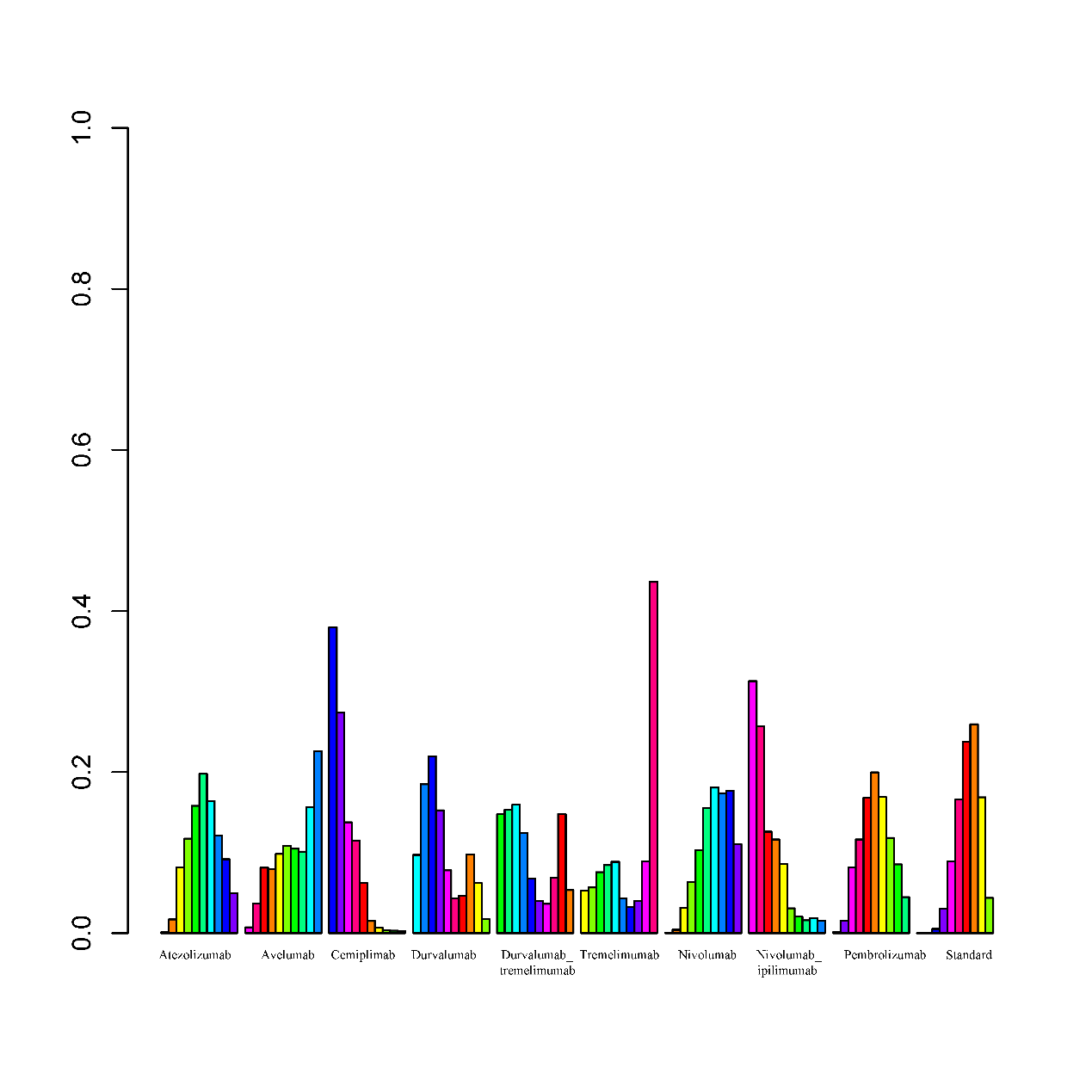


**Figure S2 Probability ranking diagram in the lung cancer subgroup for PFS.** Probability ranking diagram shows the probability of the safety of different therapies ranking the first to the last for PFS.

**
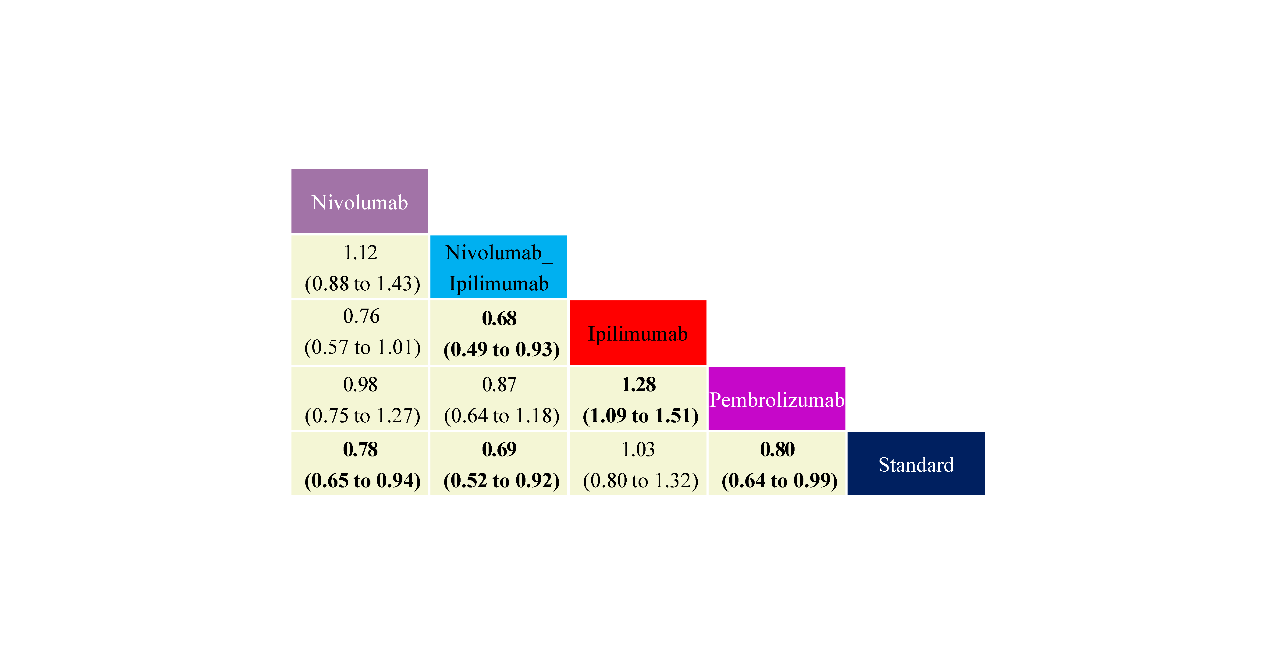
**

**Figure S3 Safety profile in the melanoma subgroup for PFS.** In the safety profile, efficacy of treatment for PFS is represented as HRs with 95% credibility intervals. All comparisons are made as column versus row. Statistically important results are in bold.


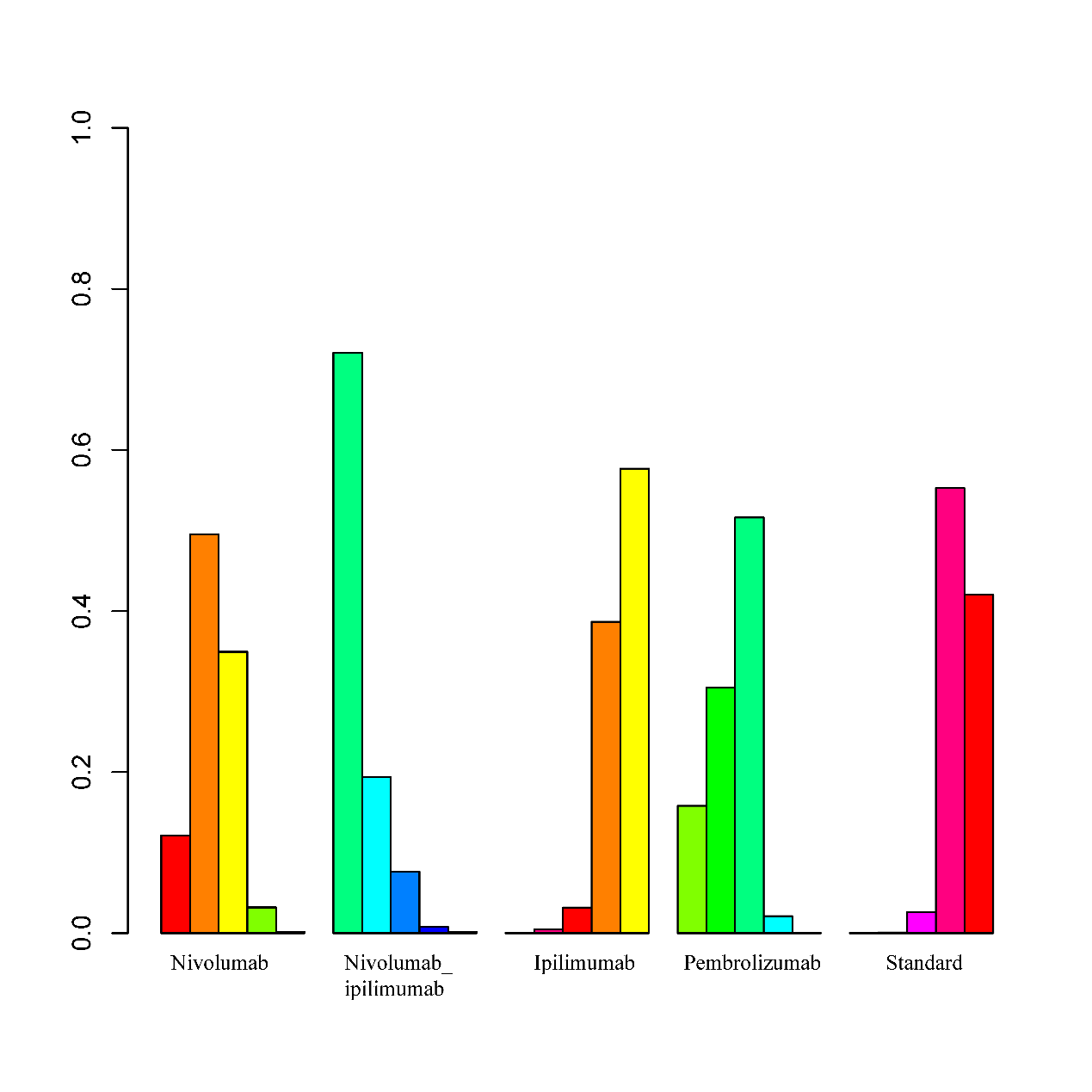


**Figure S4 Probability ranking diagram in the melanoma subgroup for PFS.** Probability ranking diagram shows the probability of the safety of different therapies ranking the first to the last for PFS.

**
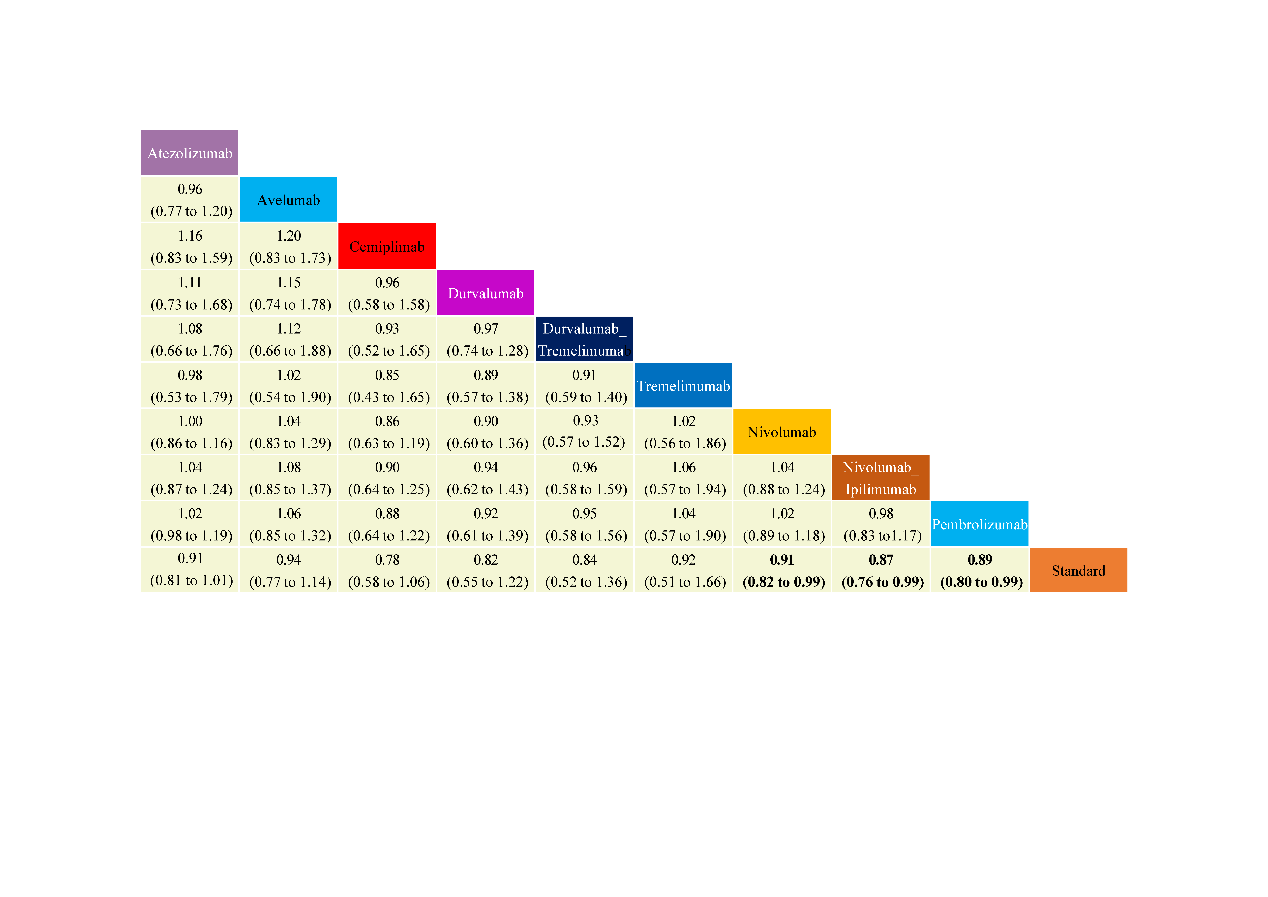
**

**Figure S5 Safety profile in the lung cancer subgroup for OS.** In the safety profile, efficacy of treatment for OS is represented as HRs with 95% credibility intervals. All comparisons are made as column versus row. Statistically important results are in bold.


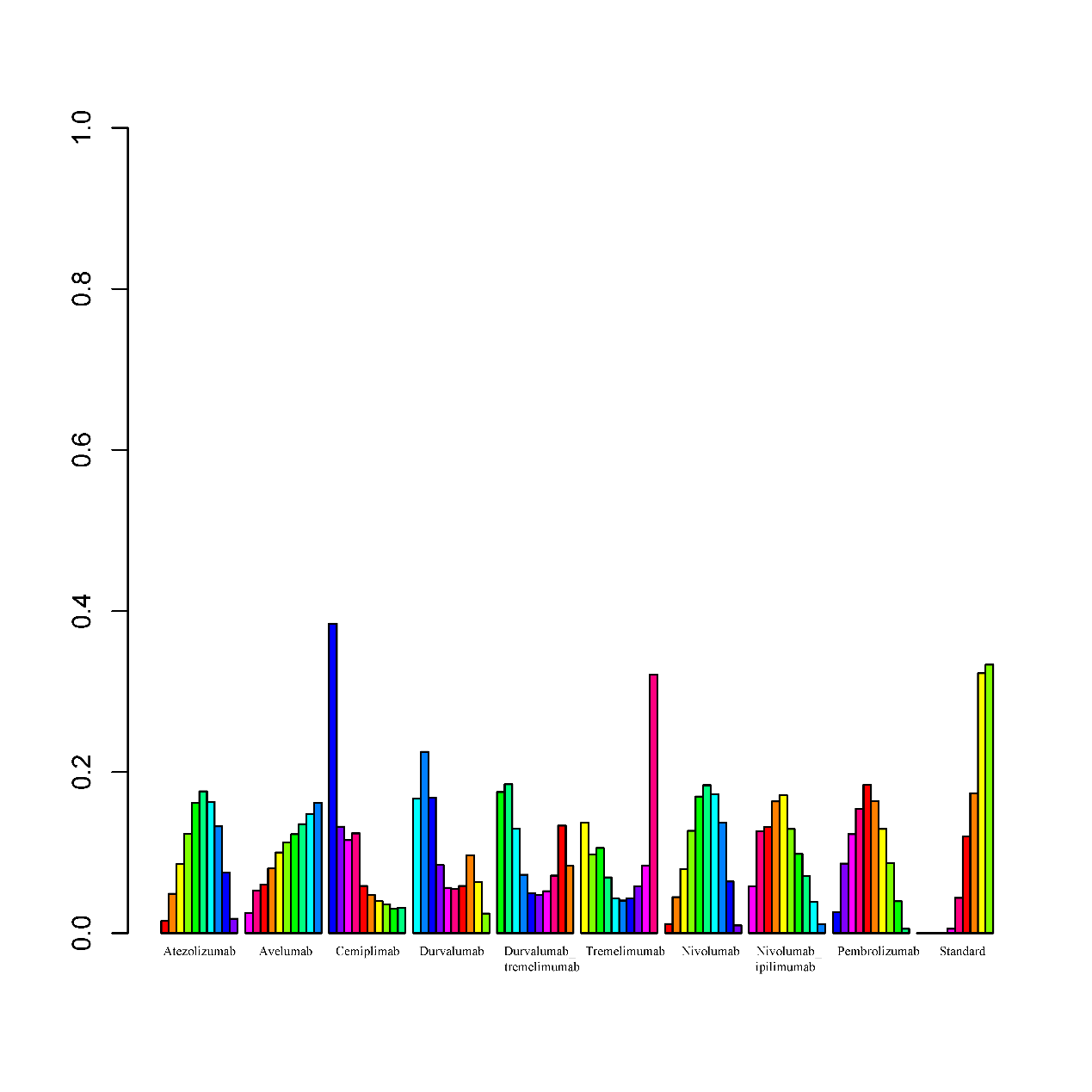


**Figure S6 Probability ranking diagram in the lung cancer subgroup for OS.** Probability ranking diagram shows the probability of the safety of different therapies ranking the first to the last for OS.

**
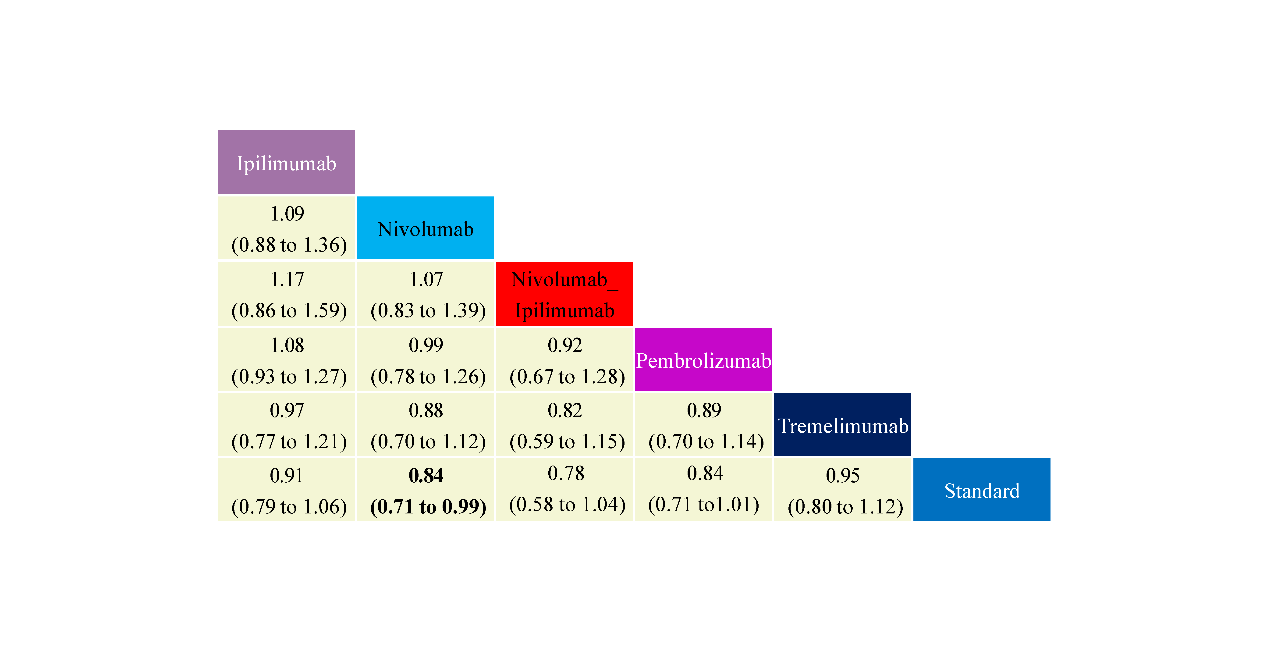
**

**Figure S7 Safety profile in the melanoma subgroup for OS.** In the safety profile, efficacy of treatment for OS is represented as HRs with 95% credibility intervals. All comparisons are made as column versus row. Statistically important results are in bold.

**
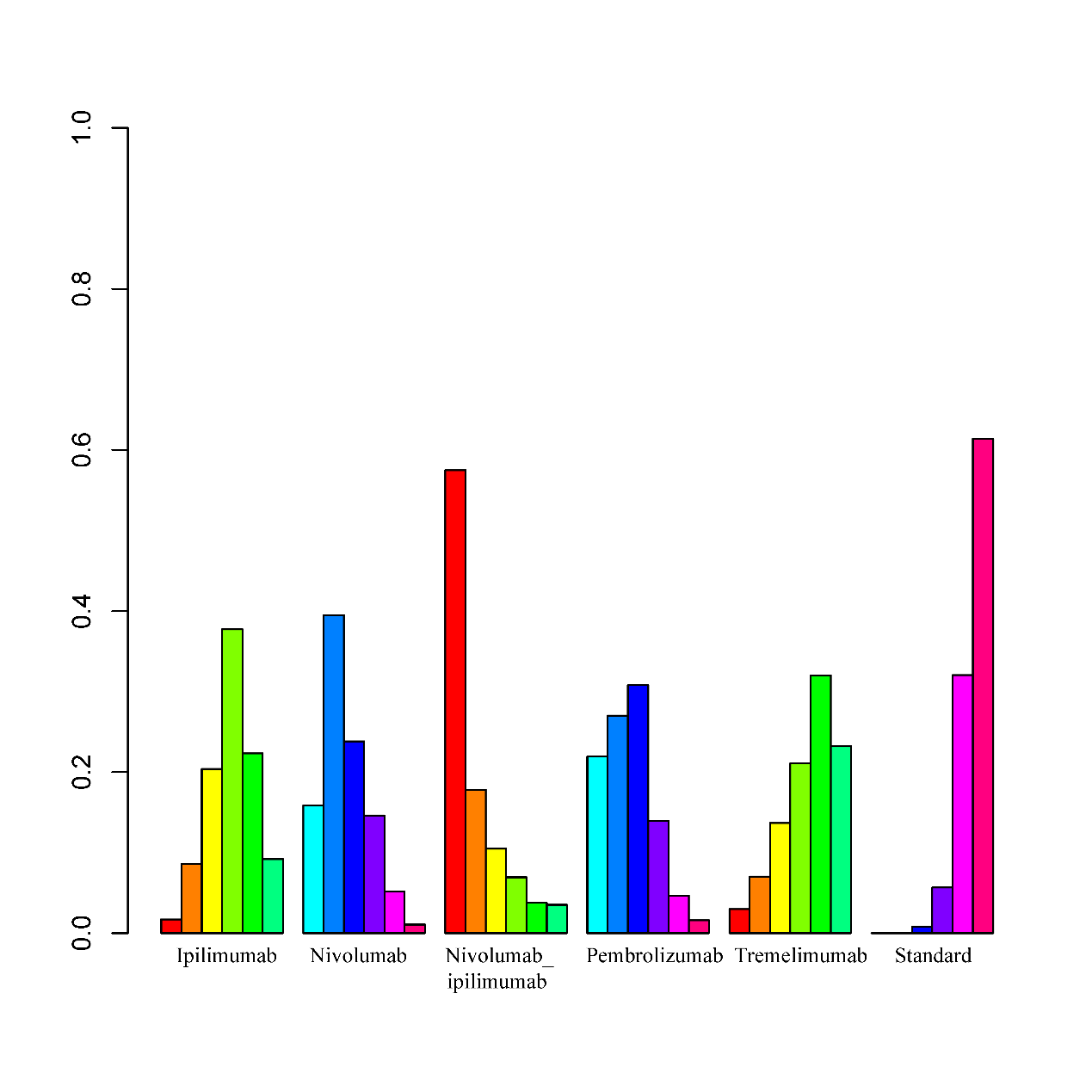
**

**Figure S8 Probability ranking diagram in the melanoma subgroup for OS.** Probability ranking diagram shows the probability of the safety of different therapies ranking the first to the last for OS.


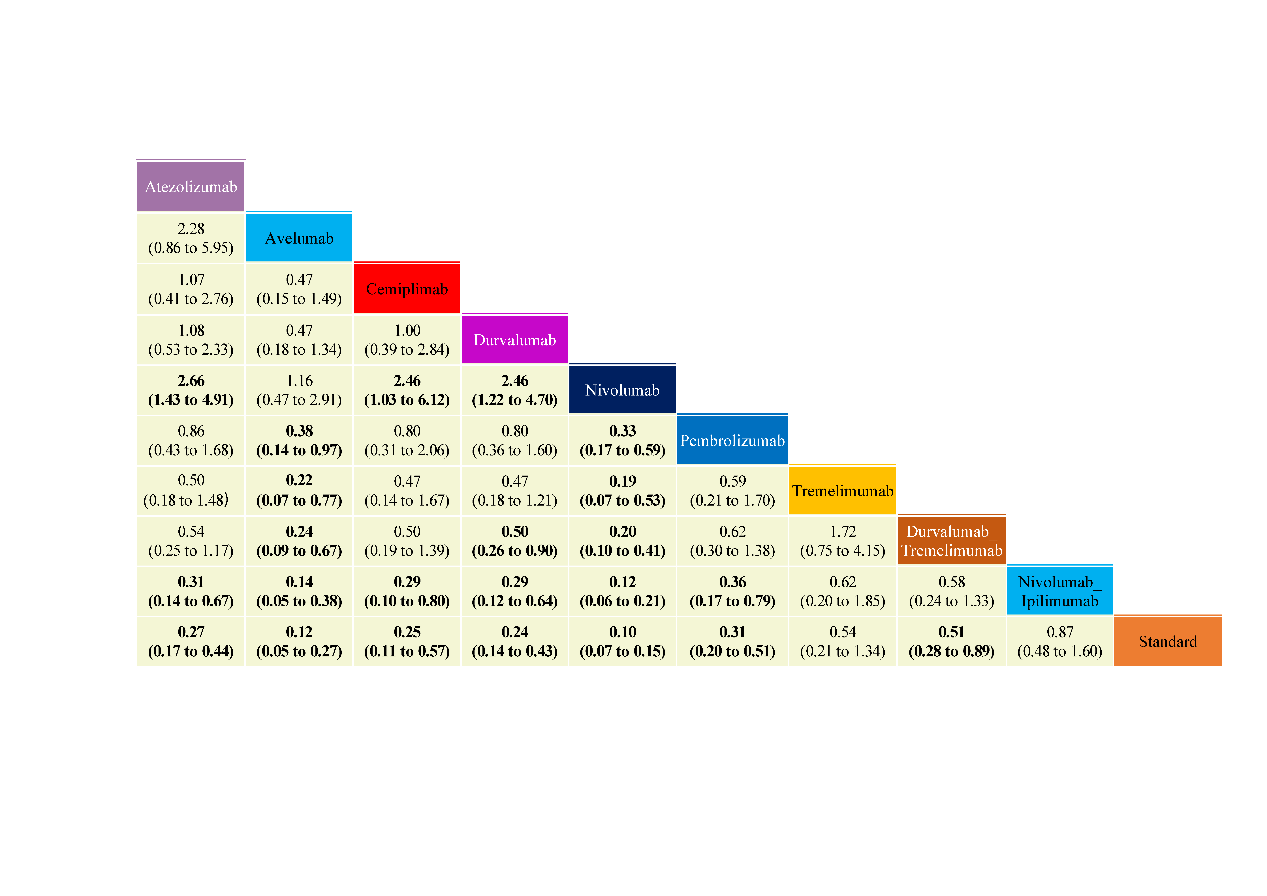


**Figure S9 Safety profile in the lung cancer subgroup for severe AEs.** In the safety profile, efficacy of treatment for grade 3-5 adverse events is represented as ORs with 95% credibility intervals. All comparisons are made as column versus row. Statistically important results are in bold.

**
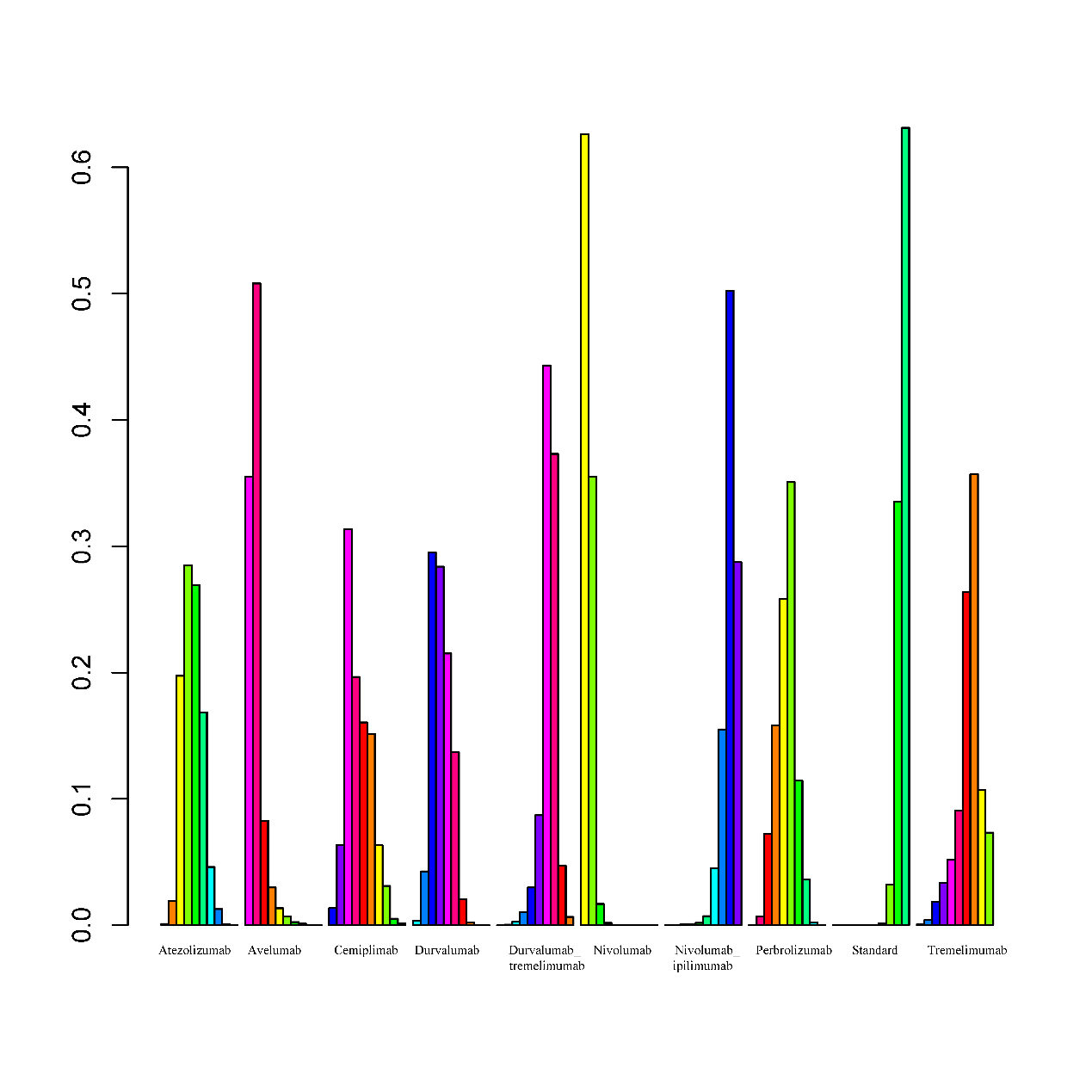
**

**Figure S10 Probability ranking diagram in the lung cancer subgroup for severe AEs.** Probability ranking diagram shows the probability of the safety of different therapies ranking the first to the last for severe AEs.

**
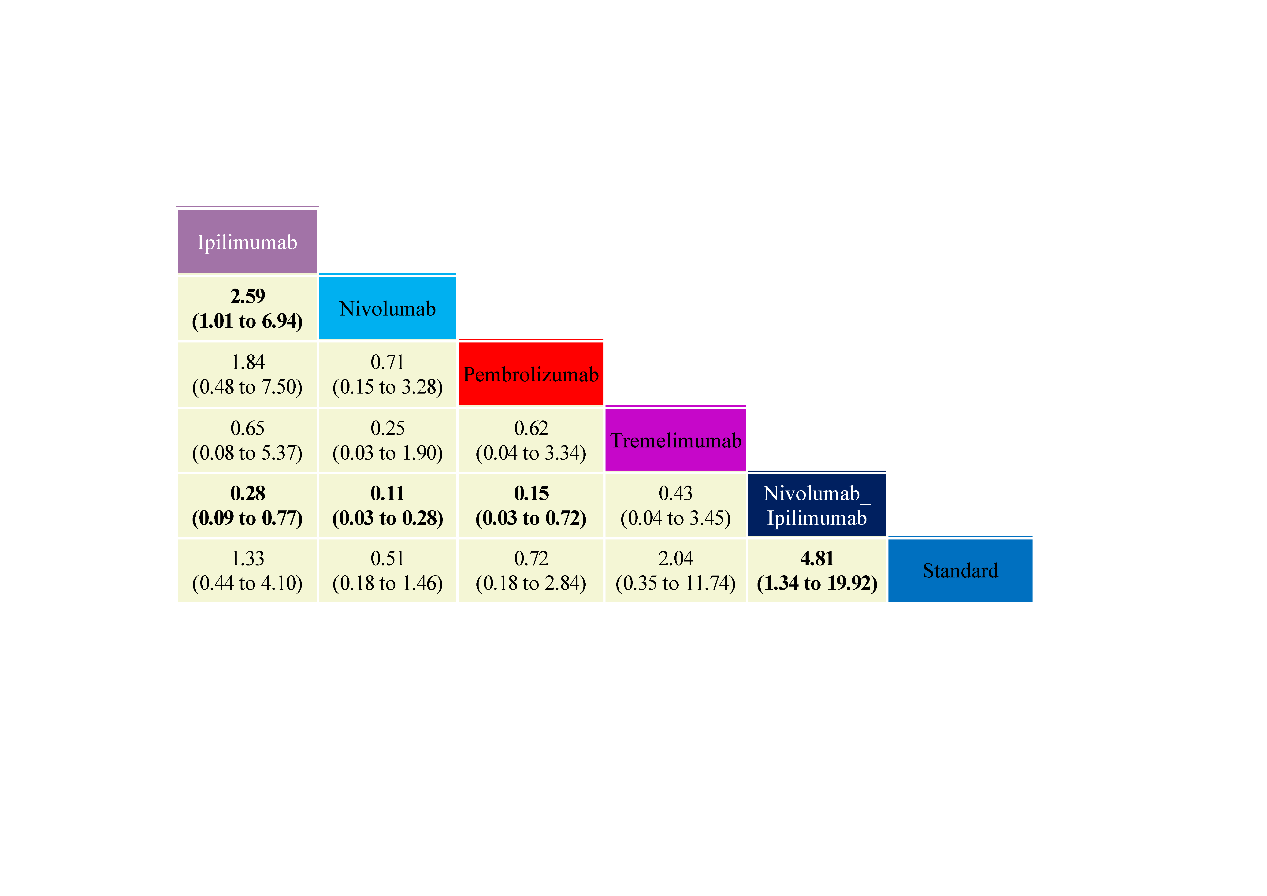
**

**Figure S11 Safety profile in the melanoma subgroup for severe AEs.** In the safety profile, efficacy of treatment for grade 3-5 adverse events is represented as ORs with 95% credibility intervals. All comparisons are made as column versus row. Statistically important results are in bold.

**
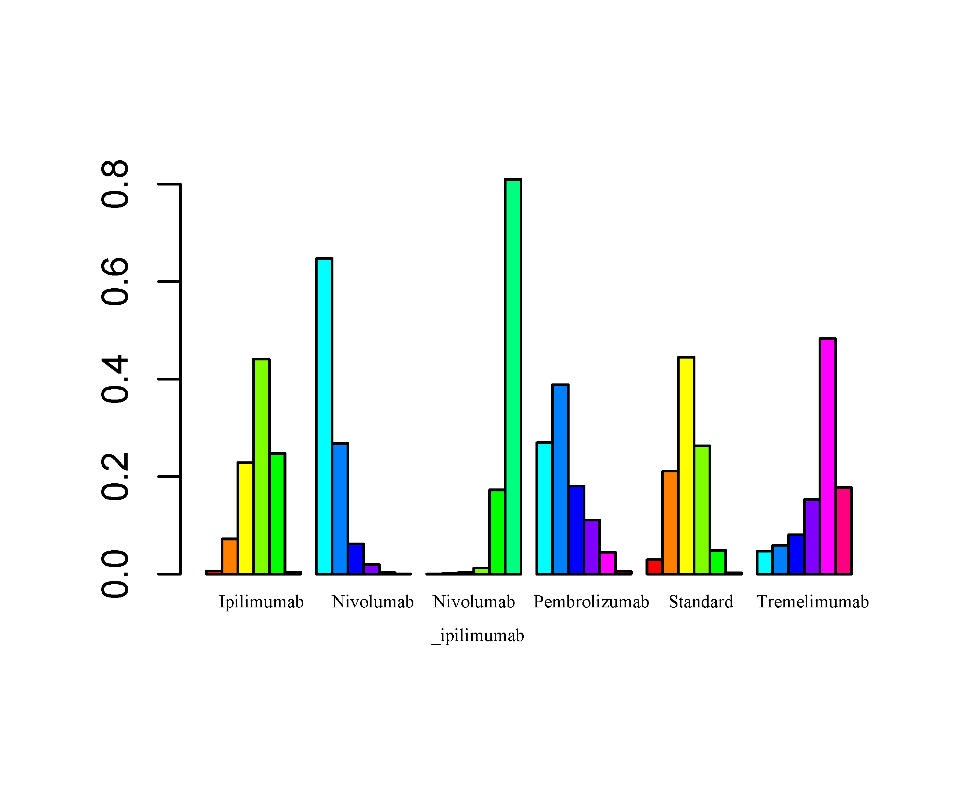
**

**Figure S12 Probability ranking diagram in the melanoma subgroup for severe AEs.** Probability ranking diagram shows the probability of the safety of different therapies ranking the first to the last for severe AEs.

**
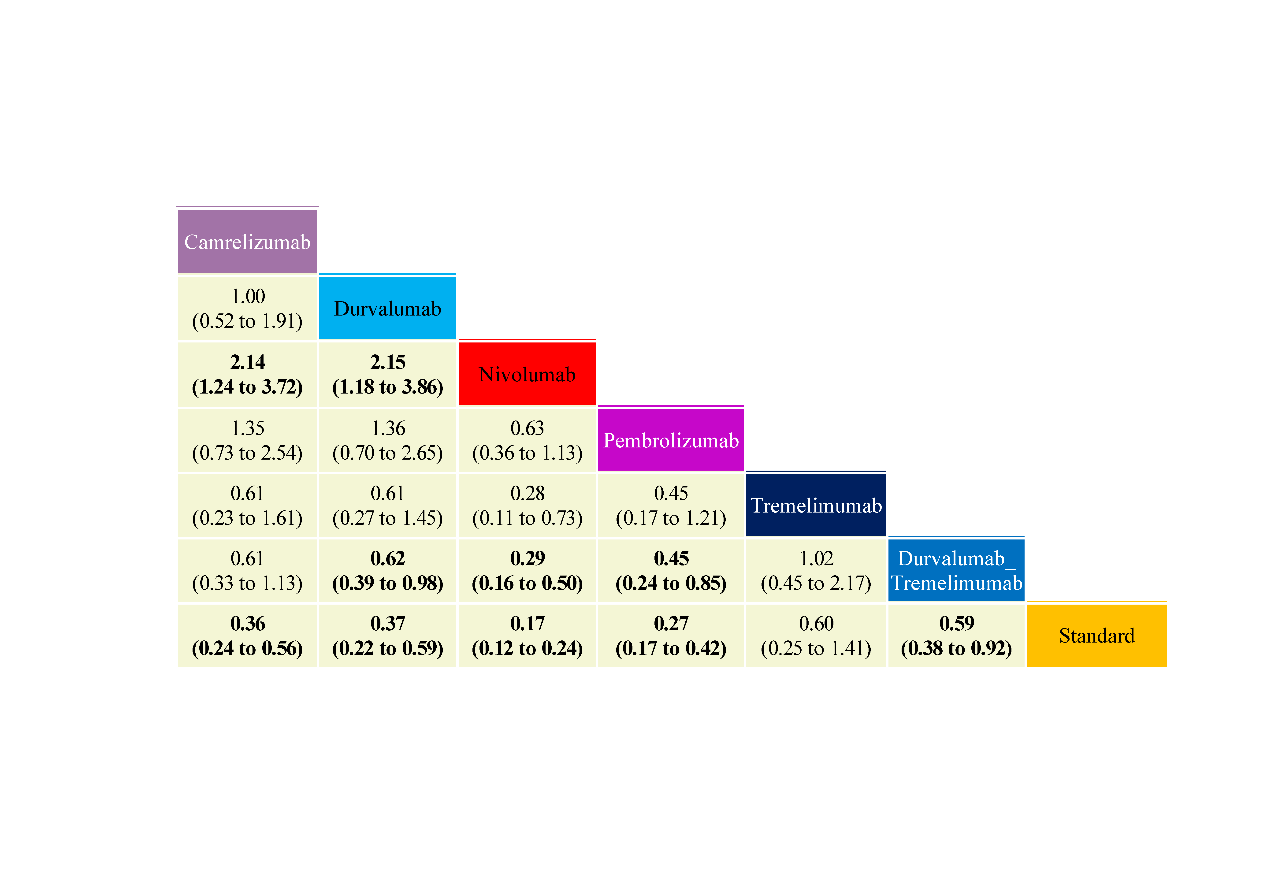
**

**Figure S13 Safety profile in the squamous cell carcinoma subgroup for severe AEs.** In the safety profile, efficacy of treatment for grade 3-5 adverse events is represented as ORs with 95% credibility intervals. All comparisons are made as column versus row. Statistically important results are in bold.

**
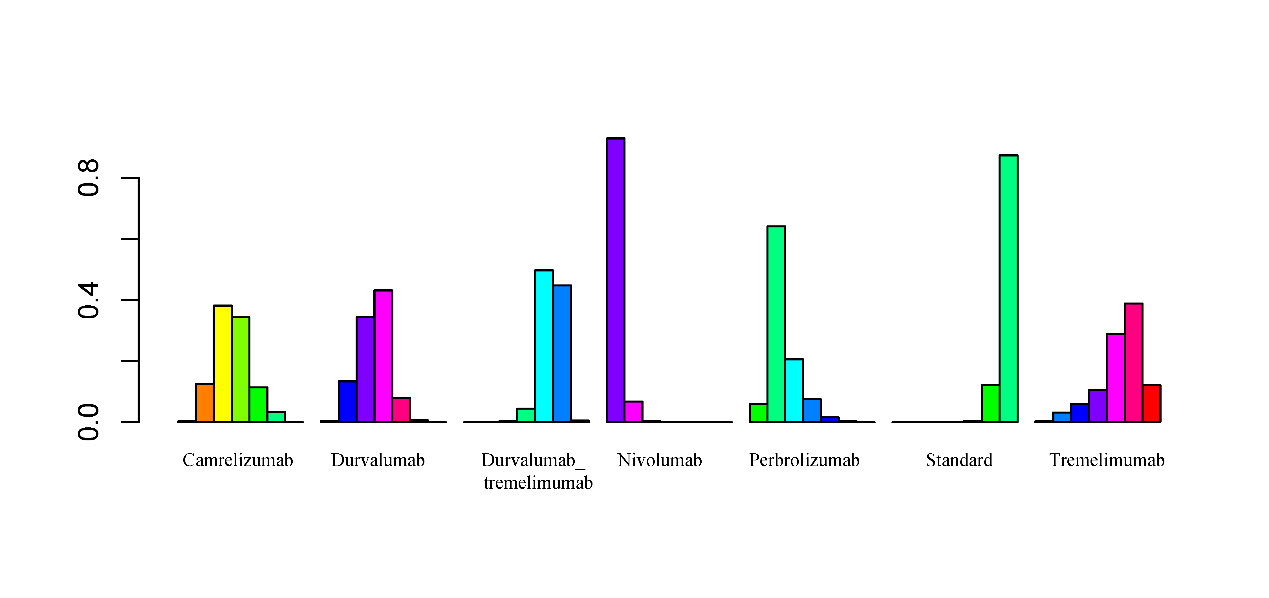
**

**Figure S14 Probability ranking diagram in the squamous cell carcinoma subgroup for severe AEs.** Probability ranking diagram shows the probability of the safety of different therapies ranking the first to the last for severe AEs.

**Table S1 Search strategy**

| Database | Keywords |
| --- | --- |
| PubMed |  |
| #1 | Cytotoxic T Lymphocyte Antigen 4 OR CTLA 4 OR CTLA-4 OR CD152 OR checkpoint inhibitor OR checkpoint blockade OR Programmed death-1 OR PD-1 OR PD1 OR Programmed death ligand-1 OR PD-L1 OR PDL1 |
| #2 | ipilimumab OR tremelimumab OR pembrolizumab OR nivolumab OR cemiplimab OR camrelizumab OR toripalimab OR tislelizumab OR spartalizumab OR atezolizumab OR avelumab OR durvalumab |
| #3 | #1 OR #2 |
| #4 | ((((((controlled clinical trial) OR randomized controlled trial)) OR random*) OR groups)) AND ((((clinical trials as topic[MeSH Terms]) OR (clinical AND trial AND topic)) OR clinical trials as topic) OR trial) |
| #5 | #3 AND #4 |
| #6 | ((randomizedcontrolledtrial[Filter]) AND (2000/1/1:2021/9/10[pdat])) AND ((clinicaltrialphaseii[Filter] OR clinicaltrialphaseiii[Filter] OR randomizedcontrolledtrial[Filter]) AND (2000/1/1:2021/9/10[pdat])) |
| #7 | #5 AND #6 |
|  |  |
| Embase |  |
| #1 | Cytotoxic T Lymphocyte Antigen 4 OR CTLA 4 OR CTLA-4 OR CD152 OR checkpoint inhibitor OR checkpoint blockade OR Programmed death-1 OR PD-1 OR PD1 OR Programmed death ligand-1 OR PD-L1 OR PDL1 |
| #2 | ipilimumab OR tremelimumab OR pembrolizumab OR nivolumab OR cemiplimab OR camrelizumab OR toripalimab OR tislelizumab OR spartalizumab OR atezolizumab OR avelumab OR durvalumab |
| #3 | #1 OR #2 |
| #4 | ('clinical trials as topic' OR trial) AND ('randomized controlled trial' OR 'controlled clinical trial' OR 'random*' OR groups)) |
| #5 | #3 AND #4 |
| #6 | [randomized controlled trial]/lim AND [2000-2021]/py |
| #7 | #5 AND #6 |
| #8 | #7 AND (‘phase 2 clinical trial’/de OR ‘phase 3 clinical trial’/de) AND ‘article’it |
|  |  |
| Cochrane Library |  |
| #1 | ipilimumab OR tremelimumab OR pembrolizumab OR nivolumab OR cemiplimab OR camrelizumab OR toripalimab OR tislelizumab OR spartalizumab OR atezolizumab OR avelumab OR durvalumab |
| #2 | Cytotoxic T Lymphocyte Antigen 4 OR CTLA 4 OR CTLA-4 OR CD152 OR checkpoint inhibitor OR checkpoint blockade OR Programmed death-1 OR PD-1 OR PD1 OR Programmed death ligand-1 OR PD-L1 OR PDL1 |
| #3 | #1 OR #2 |

**Table S1 Search strategy**

| #4 | clinical trials as topic OR trial |
| --- | --- |
| #5 | randomized controlled trial OR controlled clinical trial OR random* OR groups |
| #6 | #4 AND #5 |
| #7 | #3 AND #6 |
| #8 | with Cochrane Library publication date Between Jan 2000 and Sep 2021, in Trials |
| #9 | Source: ICTRP OR CT.gov |

**Table S2 The analysis of heterogeneity and inconsistency in terms of PFS and OS**

|  | PFS | OS |
| --- | --- | --- |
| I^2^ | 19% | 0% |
| P value heterogeneity and inconsistency | 0.14 | 0.99 |
| P value heterogeneity (within design) | 0.05 | 1.00 |
| P value inconsistency (between design) | 0.97 | 0.60 |

PFS = Progression-free survival. OS = Overall survival

**Table S3 Heterogeneity analysis of serious AEs in the network meta-analysis**

| Comparisons | Number of groups | I^2^(network) |
| --- | --- | --- |
| Sta vs Ate | 6 | 61.1% |
| Sta vs Ave | 3 | 87.1% |
| Sta vs Cam | 1 | NA |
| Sta vs Cem | 1 | NA |
| Dur+Tre vs Dur | 6 | 0.0% |
| Sta vs Dur | 6 | 84.2% |
| Tre vs Dur | 2 | 0.0% |
| Sta vs Dur+Tre | 4 | 89.0% |
| Tre vs Dur+Tre | 2 | 0.0% |
| Niv vs Ipi | 2 | 90.7% |
| Niv+Ipi vs Ipi | 2 | 91.8% |
| Pem vs Ipi | 1 | 84.3% |
| Sta vs Ipi | 2 | 90.9% |
| Niv+Ipi vs Niv | 5 | 51.1% |
| Sta vs Niv | 11 | 92.4% |
| Sta vs Niv+Ipi | 4 | 93.7% |
| Sta vs Pem | 11 | 61.3% |
| Tre vs Standard | 2 | 93.% |

AEs = Adverse events. Sta = Standard therapy. Ate = Atezolizumab. Ave = Avelumab. Cam = Camrelizumab. Cem = Cemiplimab. Dur+Tre = Durvalumab plus tremelimumab. Dur = Durvalumab. Tre = Tremelimumab. Niv = Nivolumab. Ipi = Ipilimumab. Niv+Ipi = Nivolumab plus ipilimumab. Pem = Pembrolizumab.

**Table S4 Node splitting analysis of serious AEs in the network meta-analysis**

| Nodes | Direct effect | Indirect effect | Network effect | P value |
| --- | --- | --- | --- | --- |
| Dur, Tre | 0.63 (-0.51, 1.7) | 2.0 (0.55, 3.5) | 1.1 (0.20, 1.9) | 0.13 |
| Dur, Sta | 1.30 (0.69, 1.9) | 0.00064 (-2.0, 2.0) | 1.2 (0.65, 1.8) | 0.21 |
| Ipi, Niv | -0.96 (-1.9, 0.0030) | -1.4 (-2.3, -0.55) | -1.2 (-1.8, -0.57) | 0.42 |
| Ipi, Pem | -0.16 (-1.5, 1.2) | -0.79 (-1.6, 0.0092) | -0.62 (-1.3, 0.062) | 0.42 |
| Ipi, Niv+Ipi | 1.5 (0.49, 2.4) | -0.18 (-1.0, 0.70) | 0.57 (-0.10, 1.2) | 0.01 |
| Ipi, Sta | -0.97 (-2.1, 0.088) | 0.93 (0.24, 1.6) | 0.37 (-0.25, 0.97) | 0.004 |
| Niv, Niv+Ipi | 1.8 (1.1, 2.5) | 1.6 (0.91, 2.4) | 1.8 (1.3, 2.3) | 0.75 |
| Niv, Sta | 1.5 (1.1, 2.0) | 1.6 (0.76, 2.4) | 1.6 (1.2, 1.9) | 0.93 |
| Pem, Sta | 1.0 (0.62, 1.5) | 0.42 (-1.1, 1.9) | 1.0 (0.58, 1.4) | 0.43 |
| Tre, Dur+Tre | -0.069 (-1.1, 1.0) | -1.4 (-2.9, 0.15) | -0.42 (-1.2, 0.44) | 0.17 |
| Dur+Tre, Sta | 0.79 (0.087, 1.5) | -0.26 (-1.7, 1.2) | 0.57 (-0.049, 1.2) | 0.19 |
| Niv+Ipi, Sta | 0.20 (-0.47, 0.86) | -0.64 (-1.4, 0.073) | -0.20 (-0.69, 0.30) | 0.08 |
| Tre, Sta | -0.11 (-1.1, 0.91) | 0.74 (-0.79, 2.3) | 0.15 (-0.65, 0.97) | 0.36 |

AEs = Adverse events. Dur = Durvalumab. Tre = Tremelimumab. Sta = Standard therapy. Ipi = Ipilimumab. Niv = Nivolumab. Pem = Pembrolizumab. Niv+Ipi = Nivolumab plus ipilimumab. Dur+Tre = Durvalumab plus tremelimumab.
